# Patterns of infringement, risk, and impact driven by coal mining permits in Indonesia

**DOI:** 10.1007/s13280-023-01944-y

**Published:** 2023-10-27

**Authors:** Tim T. Werner, Tessa Toumbourou, Victor Maus, Martin C. Lukas, Laura J. Sonter, Muhamad Muhdar, Rebecca K. Runting, Anthony Bebbington

**Affiliations:** 1 School of Geography, Earth and Atmospheric Sciences, University of Melbourne, 221 Bouverie Street, Carlton, VIC Australia; 2 Institute for Ecological Economics, Vienna University of economics and Business (WU), Welthandelsplatz 1, 1020 Vienna, Austria; 3 Advancing Systems Analysis Program, International Institute for Applied Systems Analysis (IIASA), Laxenburg, Austria; 4 Department of Geography, Norwegian University of Science and Technology (NTNU), 7491 Trondheim, Norway; 5 School of Earth and Environmental Sciences, The University of Queensland, St Lucia, Queensland 4072 Australia; 6 Centre for Biodiversity and Conservation Science, The University of Queensland, St Lucia, Queensland 4072 Australia; 7 Faculty of Law, Universitas Mulawarman, Jalan Sambaliung no. 1, Samarinda, 75119 Indonesia; 8 Graduate School of Geography, Clark University, 950 Main St, Worcester, MA 01610 USA

**Keywords:** Coal, GIS, Governance, Land use change, Mining, Remote sensing

## Abstract

Coal mining is known for its contributions to climate change, but its impacts on the environment and human lives near mine sites are less widely recognised. This study integrates remote sensing, GIS, stakeholder interviews and extensive review of provincial data and documents to identify patterns of infringement, risk and impact driven by coal mining expansion across East Kalimantan, Indonesia. Specifically, we map and analyse patterns of mining concessions, land clearing, water cover, human settlement, and safety risks, and link them with mining governance and regulatory infractions related to coal mining permits. We show that excessive, improper permit granting and insufficient monitoring and oversight have led to deforestation, widespread overlaps of concessions with settlements, extensive boundary and regulatory violations, lacking reclamation, and numerous deaths. As the world’s largest thermal coal exporter, Indonesia’s elevated coal infringements, risks, and impacts translate to supply chain, sustainability, and human rights concerns for global coal markets.

**Supplementary Information:**

The online version contains supplementary material available at 10.1007/s13280-023-01944-y.

## Introduction

**Fig. 1:**
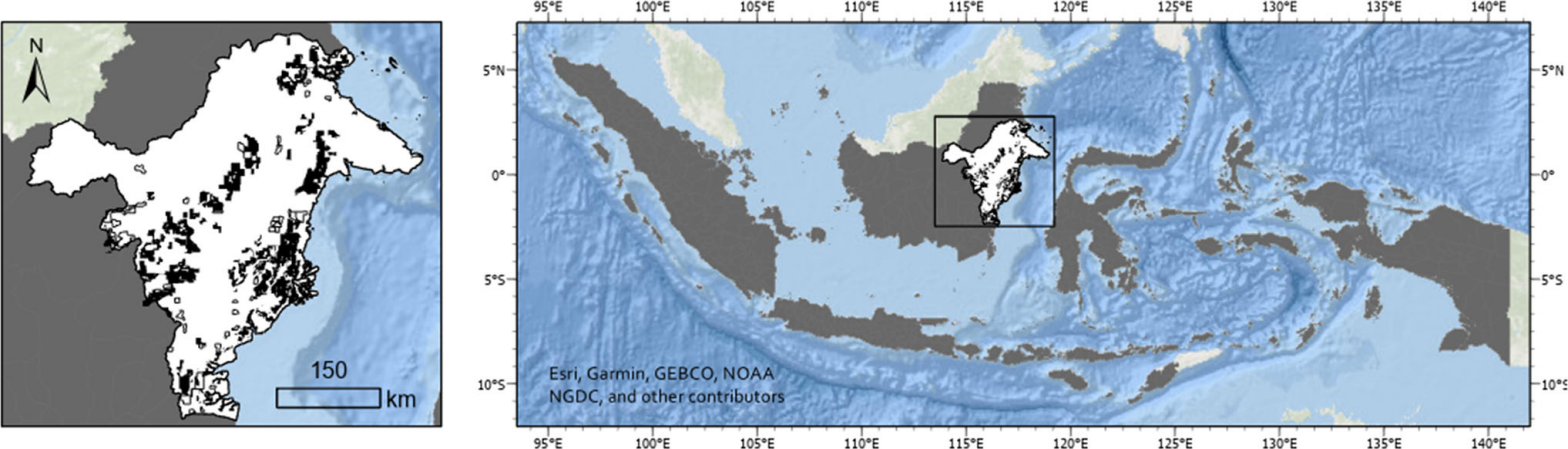
Study area of East Kalimantan (white) in Indonesia (grey), with coal mining concessions allotted within the province (black, per data source C-A, see Table S3)

The expansion of coal mining, the largest source of global energy-related carbon emissions, is driven largely by national and corporate political-economic agendas. Amid global debates about coal exports and climate change, the carbon footprint of coal mining has received greater attention (Faisal et al. 2018; Edwards 2019; Nasih et al. 2019) than the lesser studied ‘out of sight’ environmental and social impacts at sites of extraction in Indonesia (Fatah 2008; Bell and York 2012; Fünfgeld 2016). The province of East Kalimantan, covering 12.7 Mha, is the largest and most populous Indonesian province on the island of Borneo. It supports 52% of Indonesia’s total thermal coal production (Agrawal et al. 2018). Approximately 40% of the land area of the province has been allotted to open-pit coal mining (Fig. 1), with associated production supporting Indonesia to become the world’s largest exporter of thermal coal (BP 2018; IEA 2020). The province is extremely rich in endemic plant and vertebrate species (Fuller et al. 2010). Its forests, which belong to one of the world’s most significant biodiversity hotspots (Myers et al. 2000; Roos et al. 2004), have been particularly threatened by mining, logging, and plantation development (Austin et al. 2019; Giljum et al. 2022), with significant declines in coverage and quality reported over 2000–2016, a period in which mine areas more than tripled (Kiswanto et al. 2018).

East Kalimantan is Indonesia’s second most prosperous province (by GDP/capita) and social inequalities are growing (Wahyuningsih et al. 2019). The economic dominance of extractives in this province suggests that the economic benefits of extraction have not reduced inequality and given the weight of the sector in the province’s economy, have instead likely sharpened inequality.

Industrial-scale resource extraction first began in the province in the late 1960s, when the Suharto administration introduced a suite of laws that worked to secure central government control over natural resources and cultivate the forestry and coal mining industries. The devolution of authority over mining introduced in the *reformasi*-era reforms that followed the end of the Suharto regime in 1998 shifted control over mining licensing from the central to district and municipal administrations (with successive revisions in 2014 moving control to provincial governments, in 2020 to central government, and in 2022 back to provincial governments). This devolution prompted widespread and largely unconstrained licensing of coal concessions, most prominently in East Kalimantan (Fünfgeld 2016; Atteridge et al. 2018).

This expansion of coal mining has had a range of adverse impacts, including destruction and degradation of Borneo’s biodiverse ecosystems (Fuller et al. 2010), land use disputes, violence, and protracted legal struggles, mainly due to the hazards that coal mines pose to nearby communities (Dewi et al. 2005; Lucarelli 2010; Fünfgeld 2016; Hilmawan et al. 2016; Kholis et al. 2016). Coal pits in the province quickly accumulate rainwater that must be routinely pumped during mine operations, risking pollution of surrounding groundwater and drinking water resources (JATAM 2017; JATAM and Waterkeeper Alliance 2018). When operations cease, water-filled mine pits often remain as polluted, unusable areas that also present drowning hazards^1^. These hazards could be reduced by common mine rehabilitation practices, including the backfilling of coal pits with soil and overburden, and the construction of protective fencing around pit areas. However, local reports note that such works rarely proceed in East Kalimantan, and that mines are operating nearer to adjacent settlements than legally allowed (Toumbourou et al. 2020b). A tragic consequence of this is that at least 40 drownings, mostly of children, have been reported in the province’s coal pits since 2011 (Jong 2021).

Despite intimidation and violence from mafia and para-military groups that protect politico-business elites (Fünfgeld 2016), local activism against poor coal mining governance (Jorde 2013; U.S. Department of State 2015) successfully fostered the introduction of provincial laws aimed at improving regulatory oversight of East Kalimantan’s coal mines (see further explanation of these laws in supplementary section S1). These laws have led to some operating permits being revoked, and temporary moratoria in the granting of new permits. Yet, there are strong indications that conflicts, environmental impacts, and human safety challenges posed by coal mining in East Kalimantan will persist and, most likely, increase. These indications include that: (1) a large portion of the province still remains open for further coal operations, (2) coal mining remains central to the national government’s economic development and energy policy plans^2^ (Government of Indonesia 2011), (3) recent changes in federal mining legislation have sought to remove red tape and increase existing permit areas (Toumbourou et al. 2020a), and (4) following increasing issues of flooding/inundation in Jakarta, Indonesia’s President Joko Widodo announced plans to develop a new national capital city, Nusantara, in East Kalimantan, which has already begun construction (Henschke and Utama 2020; Lechner et al. 2022). Combined, these factors suggest increased risk of land use conflicts and safety hazards, as greater populations will reside in proximity to hazardous mine sites that are themselves expanding.

Research assessing the extent of coal mine areas and impacts in East Kalimantan has been constrained by limited public disclosures regarding coal mining permit areas. Issues such as forest cover (Broich et al. 2011; Abood et al. 2015), conflict due to oil palm plantation development (Abram et al. 2017), or broader threats to biodiversity (Fuller et al. 2010; Sonter et al. 2020b) have attracted more attention in the literature than mining explicitly. As such, an in-depth understanding of geographical patterns associated with coal mining has been lacking. In past work (Toumbourou et al. 2020a), we analysed the political economy dynamics surrounding coal mining in East Kalimantan, including flaws in sub-national regulations governing coal, extensive networks connecting political actors, miners and para-military organisations that inhibit public scrutiny and reform efforts, and opaque coal mining licensing processes, that has given rise to the emergence of hazardous water-filled mining pits and conflict. However, we noted that the spread of these issues and their spatial relationships remained largely unexplored.

In this study, we therefore analyse in spatial detail how these issues relate to, or are driven by, coal mining permits in East Kalimantan. We combine remote sensing, extensive GIS overlays, interviews, and analyses of provincial concession data and documents to produce and discuss maps that illustrate emerging spatial patterns of infringement, risk, and impact. In the following sections, we describe the collection, organisation and interpretation of spatial and non-spatial data that enable us to identify patterns emerging due to the allocation of coal mining permits. Further methodological details are provided in supplementary sections S2 and S3. In our results section we visualise and describe these patterns and use our analysis of local literature and interviews to help explain them. This provides a basis for broader discussions of (1) how select coal-associated risks may play out considering the substantial developments (coal and otherwise) planned for this province, and (2) possible policy remedies.

## Materials and methods

### Study design

Our analysis focused on the creation, collection, and analysis of spatial and tabular datasets on 7 aspects of coal mining and its impacts: coal permit areas, coal mine areas (i.e., areas of active mining extraction and waste disposal), village locations, urban area extent, emergence of water bodies, vegetation cover, and location of coal pit-associated fatalities in East Kalimantan. Collectively, these data enable a spatial representation of infringement and select coal-associated human and environmental risks that have notably dominated narratives around coal mining governance and conflict in this region (see Toumbourou et al. 2020b, 2022, 2023). Other effects and risks that are widely associated with mining but beyond the scope of our study include health impacts (Zhang et al. 2021; Mueller 2022), biodiversity impacts (Sonter et al. 2020a), impacts to water and air quality (Tiwari et al. 2015; Hendryx 2019), and economic flows (Ejdemo & Söderholm 2011).

**Fig. 2:**
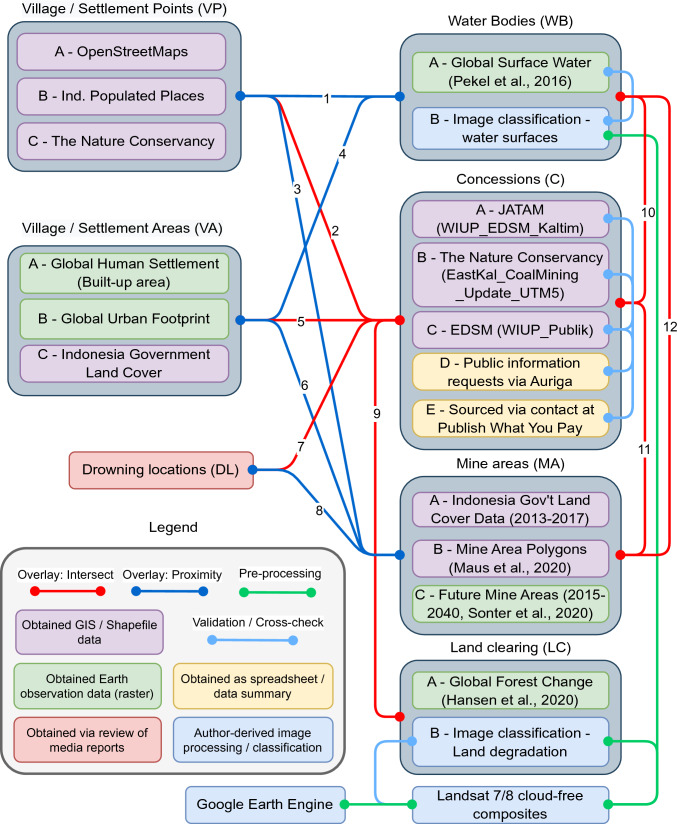
Schematic of data categories, accompanying data sources created or obtained, data formats, processes of validation, and the spatial connections explored between data categories

Data were acquired via government and academic literature sources, local media reports, non-government organisation sources, and through interviews by the authors. We also obtained, pre-processed, and analysed Landsat satellite data (2005–2020) to assess land cover and coal-associated land clearing across the province. These satellite data were chosen for their suitable spatiotemporal coverage of East Kalimantan and due to evidence of past success in the identification and analysis of mine areas with Landsat (see Werner et al. 2019). To assess the relationships between each of the above aspects, we assessed spatial intersections and proximities per Fig. 2 to build a detailed geographical profile of infringements and selected coal mining risks and impacts. As multiple and disparate data sources were accessed for each aspect, we were able to qualitatively evaluate data quality, and replicate spatial analyses between individual data sources, allowing results to be presented with uncertainty ranges. A total of 58 separate proximity or intersection spatial analyses were conducted, plus 20 intra-category cross-checks and extensive uncertainty assessments for image classification processes. The numbered links between datasets in Fig. 2 represent overlays that we refer to in our results to indicate which sources contributed to each result. Further detail on these methods and the source data are provided in the supplementary information.

We note that throughout this paper, the term ‘concession’ refers to the area where a coal permit has been issued for the purpose of exploration (denominated as an *Izin Usaha Pertambangan Explorasi*, IUP-E) or for operation (*Izin Usaha Pertambangan Operasi*, IUP-O), while ‘coal mine’ indicates the area of land impacted by a mine’s operating features, inclusive of extraction pits, waste disposal, and beneficiation/processing infrastructure areas. A concession can cover a larger area than the coal mine, and a coal mine can be developed outside a concession, albeit illegally. The term ‘village settlement’ includes hamlets (*dusun*) and main villages (*desa*), which can comprise a number of hamlets (*dusun)*.

### Identifying drowning locations and risk factors

To understand the spatial relationships between mining and safety, the locations of drownings were determined from a review of media articles and NGO reports, dating back to 2011. Where possible, photos in these articles were cross-referenced with recent imagery in Google Earth Pro. Where publicly reported, details of the incidents were noted, including the date of death, company operating the license area at the time of the incident, coordinates, notes on data sources and uncertainties, and a classification for location accuracy. High location accuracy (H) was assigned when a precise location, to the nearest pit or location within that pit, was determined from news articles and/or published reports. Medium (M) was assigned when the correct concession was identified, with a central or major pit within this concession selected. This entailed cross-referencing the reported village boundary with the boundary of the concession, and/or identifying pits nearest to the road that was reported as a landmark close to the place of the incident. Low (L) was assigned when the authors could not identify the correct concession area, or the incident took place outside of a legal concession, in which case a pit was selected nearest to the address given for the incident in local NGO reports and within the reported village boundary. Full details of drowning incidences are provided in the supplementary materials. The location of drownings was assessed spatially in relation to village locations, mine areas, and water bodies within these mine areas. The emergence of water bodies within coal mining areas was determined from Pekel et al. (2016) and validated against our own support vector machine learning image classification of our Google Earth Engine composites. Training samples for these composites were assigned by visual inspection and validated against government-sourced land-cover classification data from 2013, 2014, 2015, and 2017. We classify all water bodies within operating coal permit areas, and that have emerged since the date of the permit being granted as ‘water-filled pits.’ It is a noted source of uncertainty that coal pits themselves are not the only topographical features that fill with water. Coal mining-induced alterations to surrounding topography can subject new areas to water accumulation. Such sites may still present a hazard, are coal-associated and may not be fully distinguished in our analyses. However, the WB vs MA overlay (overlay 12, Fig 2.) indicates that the water bodies are accurately described as pits in key areas around Samarinda where drownings are recorded.

### Remote sensing image classification and uncertainty assessment

The potential scale of environmental degradation induced by mining can be assessed by land-cover mapping (Sonter et al. 2017; Werner et al. 2019; Werner et al. 2020). To explore these effects, in addition to the land-cover datasets obtained, we performed support vector machine learning image classification using training data obtained from Maus et al. (2020a, b) and through the replication of visual inspection methods described in Kiswanto et al. (2018) and Werner et al. (2020). Given that mine areas reflect similar spectral bands to other non-vegetated areas in Landsat imagery, we distinguished only between vegetated, non-vegetated, and water body areas using a 7-5-1 band combination, thereby building a profile of mine-related land clearing when overlain by coal permit areas. We used support vector machine learning image classification via ArcGIS Pro 3.1 (Esri 2023) to conduct these analyses. Additional land clearing data were obtained from Hansen et al. (2013) to support a broader view of forest loss patterns in the province.

We validated our land-cover classification following state-of-the-art accuracy assessment methods (Olofsson et al. 2013; Olofsson et al. 2014). We first ran a stratified sampling design with a standard error of expected overall accuracy equal to 0.01 to ensure that the sample size is large enough to produce sufficiently precise estimates of the area of the class. Based on the sampling design, we drew 508 random samples stratified by class, 100 non-vegetated, 308-vegetated, and 100-water. The samples were then validated independently using visual interpretation of a 10 meters spatial resolution Sentinel-2 composite. The composite was processed using Google Earth Engine platform and has the median pixel values derived from clear images acquired between 2017-01-01 and 2019-12-31. The overall accuracy of the map reached 96.2% (Table 1).

**Table 1:** Confusion matrix and accuracy metrics for 2017–2019 land-cover map based on the 508 validation points. All three classes present user’s accuracy higher than 80%—this metric is particularly high for vegetated areas (95.8%). User’s accuracy also has a relatively narrow confidence interval at 95%. Producer’s accuracy was lower for non-vegetated (52.6%) and water (55.8%) areas with a relatively wide confidence interval at 95%. These classes can be underestimated on our maps and have higher classification uncertainty than vegetated area, whose producer’s accuracy was relatively high (99.1%) with narrow confidence interval at 95%

Mapped	Reference				User’s acc. (%)	F1 Score
	Non-vegetated	Vegetated	Water	Total		
Non-vegetated	87	13	0	100	87.0 ±6.6	87.4
Vegetated	9	295	4	308	95.8 ±2.2	93.5
Water	3	15	82	100	82.0 ±7.6	88.2
Total	99	323	86	508		
Producer’s acc. (%)	52.6 ±15.8	99.1 ±0.3	55.8 ±24.2			
Overall acc.: 95.2 ±2.1%; Kappa: 0.84						

### Political economy analysis

To inform a political economy analysis supplementing the spatial analysis, we drew on key informant interviews and site observations conducted in East Kalimantan and Jakarta between December 2018 and May 2019. Twenty-three interviews (21 in East Kalimantan, plus 2 in Jakarta), were conducted by the second and seventh author in Indonesian, focussing on mine reclamation and governance. Interviewees were chosen from a range of sectors, including from national and sub-national government, non-government organisations, mining companies, and academia, to represent different perspectives of issues around coal mining. These interviews also supported previous published work (see Toumbourou et al. 2020b, wherein qualitative methods are further explained). Transcripts were translated and coded by the second author through the interview process to identify emerging codes and arrange these into themes, to ensure that data saturation was reached (that is, the same comments or themes were emerging in new interviews, Glaser et al. 1968; Guest et al. 2006). Interview data were re-analysed using content analysis to identify emergent themes to help explain the underlying political economy factors enabling the distribution of coal mining permits revealed in our spatial analysis. This was complemented by new analysis of relevant regulations and laws, environmental impact assessments, mine reclamation plans, and government agency and NGO investigation reports.

Alongside interviews, a focus group discussion was also organised, including eight participants from civil society organisations and lawyers, to explore issues around permit issuance, management and oversight of mining operations, and civil society reform efforts to improve coal mining regulation. A meeting organised at the University of Mulawarman Law Faculty and JATAM was also attended; the discussion focused on corruption and other governance issues in the extractives sector. Site observations included visits to large-scale coal mines in the Mulawarman and Kerta Buana villages of the Kutai Kartenegara district (respectively one- and two-hours’ drive north of Samarinda city), and interviews with residents and leaders to understand the impacts of mining’s expansion. At both sites, large-scale coal mines had expanded over much of both village’s land, and concessions were abutting village residences. Village leaders explained that significant portions of the population residing in these villages are transmigrant farmers from Java and Bali, who settled the area between 1979 to 1982, establishing water-intensive *padi* rice farms that rely on streams and waterways for irrigation. Interviews with farmers revealed that coal mines that caused upstream water flows had been cut off, and tailings and acidic water in waterways had significantly reduced rice and crop yields (see Toumbourou et al 2020a, b).

These qualitative investigations were used to (1) validate the selection of key data categories to consider for mapping, and (2) identify and explain what socio-political processes might underpin the relationships (illustrated in Fig. 2) assessed through spatial overlay analysis.

## Results

### Coal mining and land clearance

According to land-cover data obtained from government sources, areas of land directly occupied by coal mining activity (comprising features like pits, waste rock dumps and ponds, distinct from the broader permit areas) grew 9% from 2015 to 2017 (~ 116,844 ha to 130,625 ha) and may reach ~142,348 ha by 2040 (Sonter et al. 2020b). However, if these mine areas are delineated with simplified or buffered polygons (that is, they are mapped to include the areas between closely situated mine features), up to ~140,766 ha are already occupied across the province (Maus et al. 2020a, b).

**Fig. 3:**
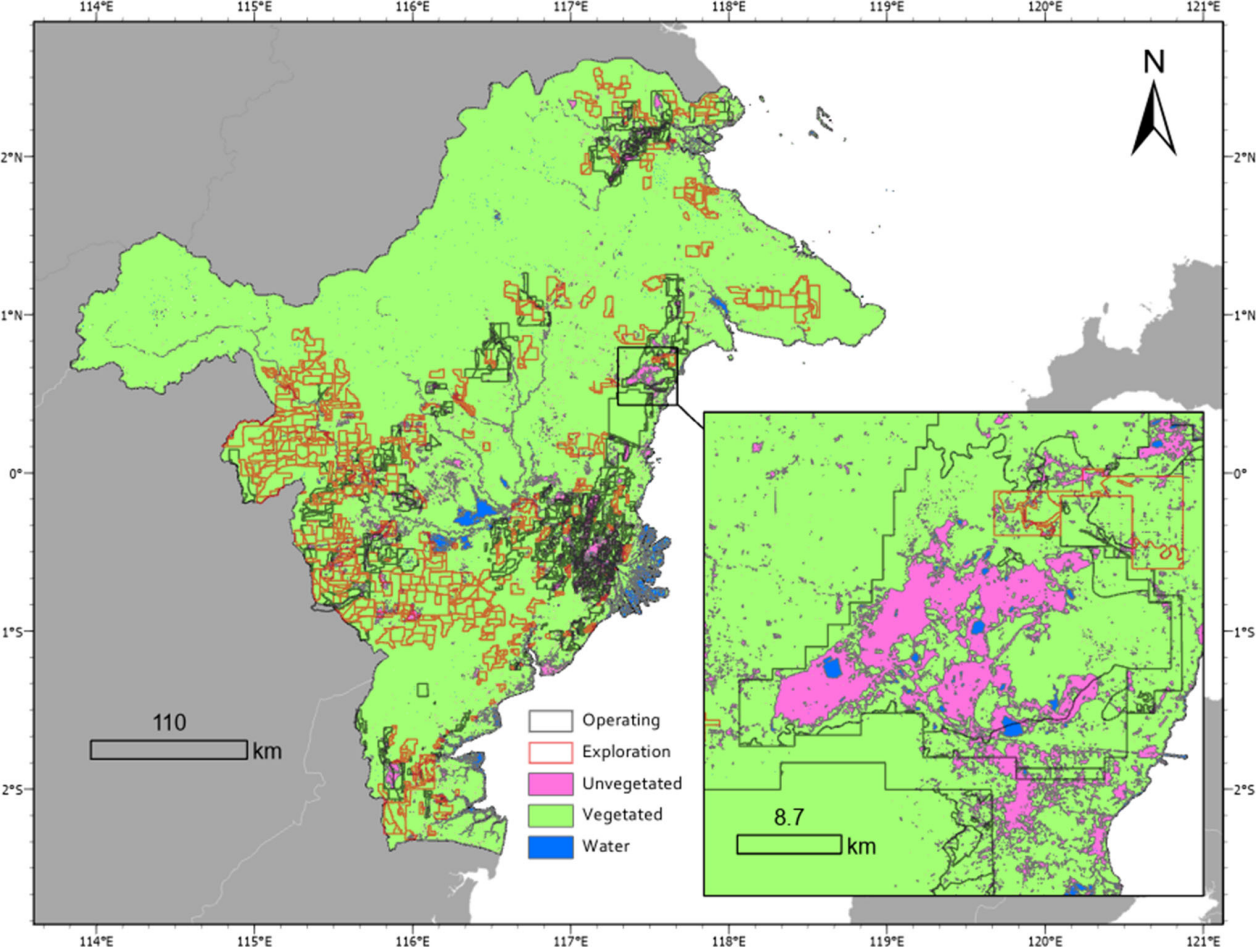
Areas classified as non-vegetated, vegetated and those with water cover, resulting from support vector machine learning classification of Landsat imagery from Period C. Concession data (outlines) from data source C–B, The Nature Conservancy, illustrate the largest potential extent of future coal-associated land clearing in red outlines

Permit area data shows that ~40% of East Kalimantan has been allotted to coal mining exploration or active operations. A breakdown of these areas for each data source can be found in supplementary section S4.2. Our satellite image analyses show that the issuance of an operating permit resulted in land clearance of 10.6-11.7% of the total concession area to date, compared to 3.5% average non-vegetated areas for the remainder of the province (overlay 9). Areas with an active operating permit had ~280% the rate of land clearing of exploration permit areas (Table 2, Fig. 3).

**Table 2:** Land-cover categories per coal concession category for the Period C composite image, using concession dataset C-A

	Operating (%)	Exploration (%)	Rest of province (%)
Non-vegetated	11.7	4.2	2.2
Vegetated	87.3	95.6	95.8
Water cover	1.0	0.2	2.0

**Fig. 4:**
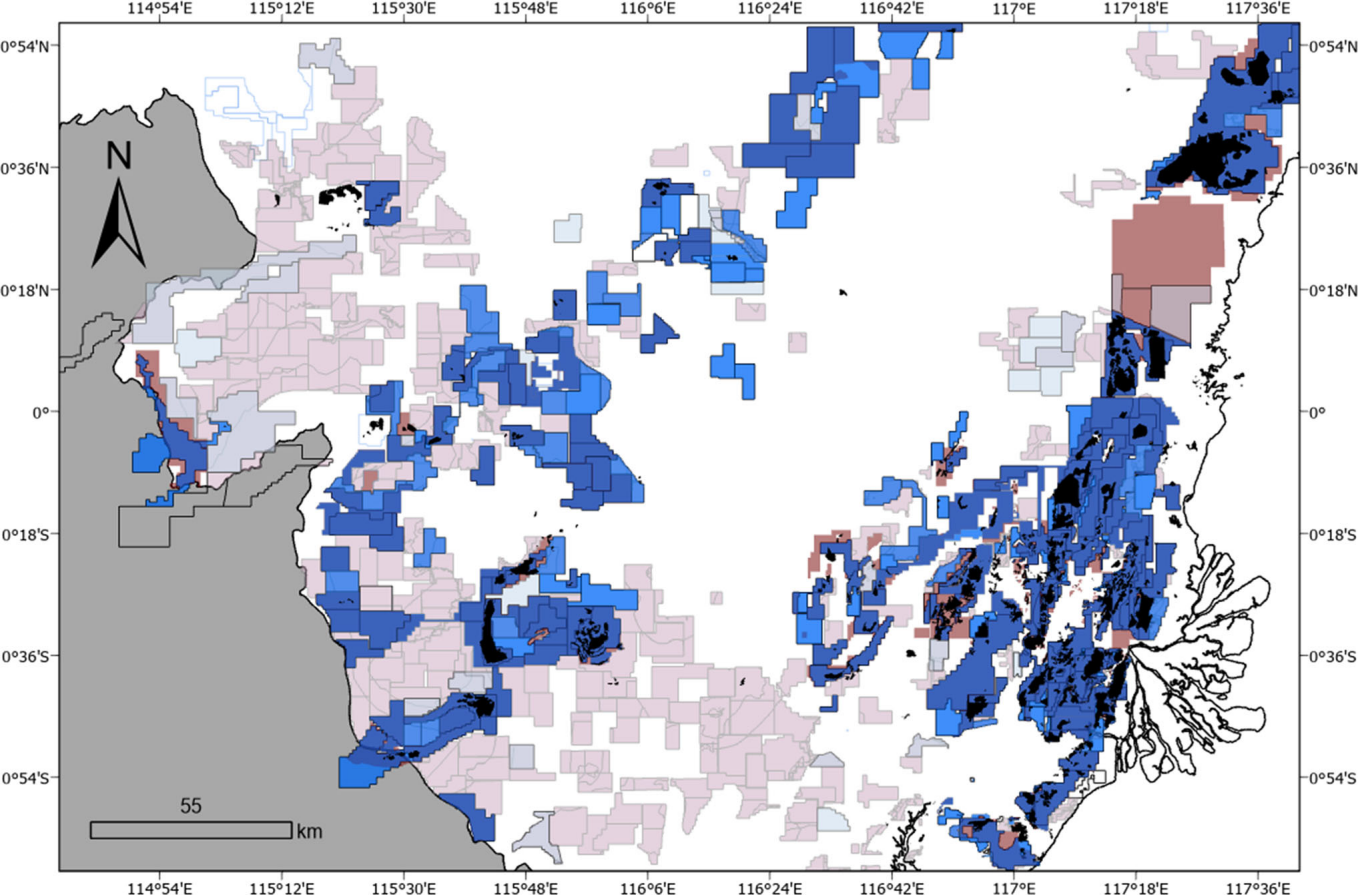
Coal mining concessions (blue = JATAM, red = The Nature Conservancy). Solid colours represent operating permit areas. Faded areas indicate mining exploration permits, illustrating the extent of mining planned for the province. Actual coal mining activity (comprising pits and associated infrastructure) is shown in solid black

It is difficult to determine the true extent of areas allocated for exploration, as concession datasets may list between 4.3 and 73.4% of permits as IUP-E, depending on the source. Further, these data differ in the number of permits, as well as their spatial boundaries, highlighting major reporting inconsistencies (Fig. 4; Table S3). However, per our review of provincial regulations, a company that holds an exploration permit (IUP-E) and that has complied with license conditions has a legally binding automatic priority right to apply for an operating permit (IUP-O, Indonesia Mining Institute 2018), suggesting that regardless of which permit data source is considered, there is still considerable capacity for expanded land clearing in the future. Interview respondents indicated that a large number of exploration permits may be in part explained by common practices of land banking, in which local business actors obtain mining permits that they sell to third parties when the development of new mines appears economically attractive. If all the current exploration permits are granted operating rights and subsequently mined, we can expect that at minimum 8,371 ha, up to potentially 178,152 ha will be cleared (applying the land clearing rates of current operational sites). This large range is due to the significant range of exploration areas reported between The Nature Conservancy (high end) and the Indonesian Ministry of Energy and Mineral Resources (low end, Table S3).

### Boundary and regulatory violations of coal mining

**Fig. 5:**
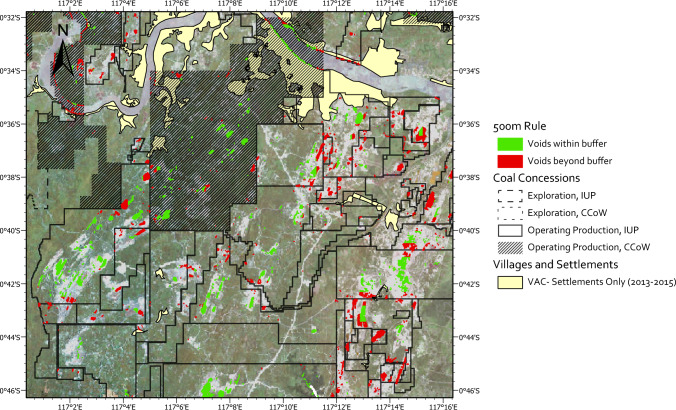
‘500 m rule’ violations. Indicates the presence of voids situated more than 500 m (green) or less than 500 m (red) of the coal permit boundaries, based on JATAM (CA) coal concession data, and satellite image classification of water-filled voids within operating permit areas. Settlement areas (yellow) ca. 2014, around the time that many of the IUP permits were granted, are also shown

We used analysis of possible regulatory infringements as a broad indicator of environmental, social and safety risks posed by coal mines and of the degree of effectiveness of resource governance. We found regulatory infringements to be wide-ranging across the province, and not constrained to particular companies or areas (Fig. 5). Such infringements were evident across all the disparate concession datasets (Table S7). Taking only government-derived data as an example (overlay 11), 16% of mine areas sit outside of permit areas, constituting 277 violations, covering ~ 21,058 ha. This suggests that previous studies in East Kalimantan may have underestimated the impacts of coal mining on forest loss by only examining forest loss within coal concessions (Abood et al. 2015).

In addition to adherence to concession boundaries, the Ministry of Environment Decree (no. 4/2012 on environmentally responsible indicators for open pit mining operations or activities) states that the distance from the edge of a mine void must be at least 500 m from an IUP concession boundary if it borders with a settlement^3^. Figure 5 illustrates the extent to which such guidelines are adhered to, with red areas showing voids within 500m of permit borders, and yellow areas showing settlement areas around the time of permit granting. As much as 33% of coal voids were situated within 500m of any permit boundary (red). It appears that some permit boundaries as visualised in Fig. 5 are too small, and too closely located to settlements to even allow the presence of any voids according to these guidelines.

Compounding these risks, an average of 26% of settlements (measured as points) in East Kalimantan had some overlap with a mining concession (Fig. S9, overlay 2). Data on concessions and settlements (measured as areas) from 2007 to 2015 indicated that this overlap constituted 14.2% (σ = 3.6%) of the area of established villages and urban settlements (Table S6). These findings are consistent with site observations and reports that coal mines have impinged on village residential areas, even so much as to have coal pits destabilising the foundations of adjacent homes (Apriando 2018).

A key mechanism introduced to tackle infringements in the mining sector was ‘Clean and Clear’ certification, a desk-based assessment of permit holders’ compliance with mining and environment related laws (see supplementary section S1 for further detail on these certifications). Our data shows that achieving this certification did not statistically reduce the number of boundary violations, nor reduce the number of fatalities, with ~51% of reported drownings occurring in areas in which permits were deemed ‘Clean and Clear’.

### Coal ponds and risks to human life

We identified the location of all but two of the 40 (at the time of writing) coal mine drownings recorded in media articles. Eighteen drowning locations (noting that some locations hosted multiple drownings) highlight that smaller pits located at the periphery of mine concessions and adjacent to roads and housing were equally as hazardous as larger coal pits at the centre of mine operations. Twenty of the reported drownings occurred in locations where the mine concession areas overlayed a village. Four of the total 40 identified drownings were associated with water bodies outside concession areas (overlay 7).

**Fig. 6:**
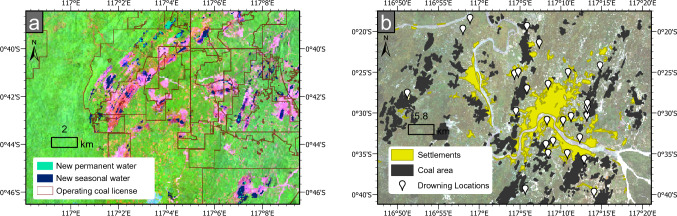
The extent of mine-associated water bodies and drowning hazards across the province. **a** False-colour composite of Landsat-8 surface reflectance imagery (7-5-1 bands, ca. 2019), highlighting non-vegetated mine areas (pink) south of Samarinda, overlayed by operating coal license areas, and newly emerged seasonal and permanent water bodies associated with mining activities. **b** Samarinda area, the location of most drownings since 2011 amid interposing settlement and coal mine areas (as at 2017). Details of drownings and their positional accuracies are provided in Table S8

Analysis of surface water emergence via satellite data (Fig. 6a showed that 27.6–27.7 km^2^ of new permanent and 90.1–112.8 km^2^ of new seasonal water bodies have emerged within operating mine permit areas (overlay 10), and notably after these permits were granted. Visual inspection methods per Werner et al. (2020) confirm that these water bodies are indeed coal pits, highlighting improper dewatering of active pits, and insufficient backfilling of inactive pits. Operating coal production areas have 570% of the water cover of exploration areas, reflecting the presence of rain-filled mine pits. Twenty-five drownings occurred in coal pits in the Samarinda municipal area, which is also where settlements and coal pits appear to overlap extensively (see Fig. 6b). Across the province, 21.6-24.8% of village settlements are immediately adjacent (that is, they directly border) surface water in operating coal permit areas [overlay 1 verified against Pekel et al. 2016), with a similar proportion directly bordering operating mine features (18.4–21.6%, overlay 3)].

Some interviewees noted that water-filled mine pits are not universally despised, as some residents use them as a source of water when they otherwise have no access to clean, municipal water (such as where mining has disrupted their former irrigation and drinking water supply). Mine pits have also been used for small-scale commercial or subsistence purposes, such as farming prawns and fish. Several drownings are linked to the use of these water bodies for various non-mining purposes, raising questions about the direct responsibility of mine operators.

## Discussion

Combined, the identified patterns of land clearing, safety hazards, and regulatory breaches point to underlying problems with mining governance in East Kalimantan and Indonesia broadly. For context to this governance, a detailed timeline of coal mining legislation and descriptions of accompanying permit rules is provided in supplementary section S1.

Our findings of increased mining-induced land clearing corroborate and are compounded by previous research (Sonter et al. 2017; Bebbington et al. 2018; Sonter et al. 2020a; Marimón et al. 2021), which shows that mines may induce secondary clearing well beyond their direct features, particularly due to the need for associated infrastructure and roads. Additionally, existing mine areas are also growing in scale. The noted differences in current mine areas between map datasets highlight a need to distinguish between areas currently showing evidence of clearing in satellite data, versus areas that may have been directly or indirectly impacted by this clearing, although the latter is often difficult to determine from satellite imagery alone.

As shown in previous work (Toumbourou et al. 2022) and by Abood et al. (2015), there is frequent overlap between mining, oil palm and other industrial concessions. This adds some uncertainty in analysing the relative contributions to forest loss from the region’s different industries. Thus, while we find mining induces increased land clearing rates in and around mine concession areas, the influence of other co-located industries remains important. Hence, land clearing as assessed in this study should be viewed as a component of a broader political-economic system that drives land clearing.

Conflicts, continued deaths, and civil society pressure have induced governance responses in recent years. For example, on 25 January 2013, the East Kalimantan governor ostensibly put a temporary freeze on the granting of new permit areas. However, a review of our concession metadata has shown that permits continued to be issued even when a moratorium was in place, with two of the licensing datasets comprising licenses dated well beyond this date.^4^ The President of Indonesia also issued a moratorium on the granting of mining permits in April 2016. Licenses also appear to have been issued or renewed following this date, however the extent that such permits were entirely new, or simply renewed, could not be determined. A major reason for this continued permitting is likely that these moratoria have not been accompanied by implementing regulations (Fünfgeld 2019), and there appear to be no actions taken against administrators that issue permits in violation of existing regulations.

While Toumbourou et al. (2020b) highlighted how provincial regulations emerged to mandate reclamation and post-mining clean-up of coal mining areas, our satellite data indicate that such activity has been limited. Respondents reported that mine permitting practices involved concessions being issued without consideration of natural landscape features or human settlements. The various violations that our data reveal show that the lines between what we might term ‘legal’ or ‘illegal’ mining are blurred in East Kalimantan. The violations documented in this paper are enabled by a number of factors, including: (1) a lack of any single detailed cadastral map of landscape features, human settlements, and land use licenses (Martono et al. 2021); (2) poor record keeping practices and a lack of cross-government agency information sharing (horizontally between departments and vertically between levels of government)^5^ in relation to land use licensing (Venugopal 2014; Yanuardi et al. 2021); (3) insufficient budget and inadequate staffing dedicated to monitoring and oversight (including addressing complaints and grievances); (4) a lack of regulatory clarity mandating the refilling of mine pits on concessions (Toumbourou et al. 2020b); and (5) challenges with law enforcement and corruption.

Each factor makes it difficult for operators, state monitoring and oversight agencies, law enforcement, and civil society observers to interpret licensing laws and consequently to monitor and enforce mining license adherence. However, it cannot be assumed that all actors have an interest in doing so. Hence, these factors simultaneously also create many possibilities for government representatives and administrative staff to contribute to illicit license granting and for operators to proceed with violations and take advantage of weaknesses and gaps in the overall regulatory system. Further aggravating these factors is the reality that royalties and rents from coal provide an important source of central and sub-national government revenues and sustain national politico-business elites, hence strong incentives remain in place to continue Indonesia’s reliance on poorly regulated coal extraction (Jakob et al. 2020; Ordonez et al. 2021).

In July 2020, Indonesia revised its mining legislation with the 2020 Mining Law. This law shifted authority over mining from provincial governments to the central government. Yet, a lack of clarity over responsibility for different tasks associated with mining oversight remains with this law. The law does also not specify any explicit requirement for refilling of voids. Instead, it merely mandates their “management”.

### Data limitations

While we have sought to maintain sufficient grounding of our geographical data through extensive review of provincial literature and local interviews, we note that there is a general risk of using GIS to represent overly simplified depictions of areas undergoing environmental or social conflicts (Spiegel et al. 2012). Our aim here has not been to produce maps of crime to be enforced upon Indonesia’s coal industry, but rather to identify patterns of landscape change and risk that may be useful in informing future development plans and mining governance reforms. The maps produced are also subject to several uncertainties stemming from limited transparency and availability of geospatial data, which hinders more precise and detailed socio-economic analysis of coal mining expansion across East Kalimantan. One such uncertainty is due to the incomplete detail in, and inconsistencies among, different mining permit databases. Each database included different detail; for example, only one of the three databases included the date of permit issuance and permit expiry. The GPS locations marking concession boundaries for each permit also differ between databases. As these same databases are relied on by government regulators for monitoring mining operations, this is a major obstacle to the state’s ability to monitor mining operations and enforce sanctions. Other data categories also exhibited variation; however, the inclusion of multiple variations of data categories, e.g., multiple sources of village point data, and settlement polygon data, has enabled us to validate results and present some uncertainty ranges. Generally, there was good agreement between datasets on human settlement areas, water bodies, and coal mining activity, but concession area datasets showed high spatial and tabular variation (such that attempts to link or combine datasets were not productive, see supplementary section S4.2).

## Conclusions

Integrating a range of spatial and qualitative data, we have produced a geographical profile of coal mining in East Kalimantan that advances the knowledge of the nature, extent, and spatial patterns of infringement and social-environmental impacts and risks. Our analysis documents elevated land clearing, limited adherence to regulatory boundaries, close proximities of mines to human settlements, the emergence of water bodies in coal mine areas, related human deaths, and lacking land rehabilitation. These issues are present throughout the province, indicating that risks extend well beyond the Samarinda area that has to date been the focus of news reports. While coal mining causes a wide range of interconnected social and ecological impacts, our study highlights a particularly devastating aspect—the alarming incidences of deaths by drowning in mine pits. We consider this as a poignant illustration of the severe and harmful impacts coal mining is inflicting on communities.

It is likely that without additional interventions, further coal mining expansion, lacking reclamation, and the population growth induced by the development of Nusantara will exacerbate many of the risks identified. Indeed, the administrative area of Nusantara itself may compete with established coal and oil palm interests, owing to considerable overlap with current concession areas (this is explored in Fig S11, see also Lechner et al. 2022 and Teo et al. 2020). However, it remains to be seen whether coal activities will be permitted to operate in this area in the same way as has been the case in the remainder of the province.

We found that past attempts to improve regulation, such as the ‘Clean and Clear’ desk-based audit mechanism are insufficient to monitor operations’ compliance to environmental and license laws. To be effective, monitoring ought to include regular field checks and independently produced maps of current mining operations to ensure that concession maps reflect the reality of mining companies’ operations, as well as of progress towards reclamation and post-mining clean-up. There is an urgent need for government to curtail the issuance of coal mining licenses and to clarify and enforce regulations around all aspects of mining, from designation of mining areas to procedures for licensing and reclamation obligations.

As a member of the Extractive Industries Transparency Initiative (EITI), Indonesia has made some formal commitments to improving reporting mechanisms. A consistent and comprehensive mining cadastre system would be an important basis for improved monitoring and oversight of all aspects of mine permitting, from issuance through to reclamation post-operation, and help to avoid the overlapping of licenses or inappropriate land zoning with natural features or human settlements. Such a cadastre should also inform spatial planning processes to ensure that mining is not allocated in (close proximity to) areas where human settlements exist or are planned. The ‘One Map Policy’ has sought to address precisely this gap (Kurniawan 2016), however at the time of writing, the cadastral issues affecting coal mining remain unresolved. Through support, incentives, and sanctions, coal-importing countries and development actors should demand improved mining governance and foster initiatives like the One Map Policy to push and assist government agencies to improve their data management and spatial analysis capacities and enhance coordination across government agencies, to ensure land permitting does not continue to threaten human lives and the environment.

The present modes of coal mining in East Kalimantan result from and illustrate substantial regulatory shortcomings of the state administrative system and problematic political-economic relations. Their environmental and humanitarian costs and associated injustices render framings of coal mining as contribution to national and regional economic and human development inappropriate. Despite high levels of coal production, coal and lignite mining contributed a mere 2.33% of Indonesia’s GDP in 2019 (Badan Pusat Statistik 2020)—revenue based on illegitimate permits and modes of operation. It benefits a few and comes at a high price. Our maps depict zones of infringement and sacrifice, in which local communities bear the negative externalities of coal mining, while the benefits are mostly captured by national and regional government, political-economic elites, and importers. Given that much of the coal extracted from East Kalimantan is destined for export, responsibility for these zones of infringement and sacrifice and corresponding needs for action extend globally. The modes of coal mining and governance of the world’s largest coal exporter and corresponding sustainability and human rights issues deserve international attention and political action.

### Supplementary Information

Below is the link to the electronic supplementary material.

Supplementary file1 (PDF 1740 kb)

## Footnotes

1 Contaminated water is an additional source of human and environmental health risk arising from abandoned coal pits. Associated health impacts may be substantial and warrant further research.

2 Indonesia’s commitment to a Just Energy Transition Partnership may change this somewhat, though initial indications are that coal will continue as a core part of the country’s energy strategy for two to three decades at least.

3 This rule is also stated in the Ministry of Energy and Mineral Resources (ESDM) regulation no. 1827 K/30/MEM/2018 about the technical guidelines for the implementation of mining.

4 On 25 January 2013, the East Kalimantan Governor Awang Faroek Ishak issued a circulation letter (Surat Edaran no. 180/1375-HK/2013) requiring district heads and mayors across East Kalimantan stop issuing new mining, logging, and plantation licenses, and ordering an evaluation of existing licenses’ compliance with regulations (though the moratorium allowed companies that had already been granted licenses to continue their operations [Toumbourou et al. 2020b]). Subsequently in April 2015, and following the Corruption Eradication Commission's (KPK) investigation of mining corruption in East Kalimantan, the provincial governor released a regulation in April 2015 (PerGub no. 17/2015) requiring that relevant East Kalimantan agencies evaluate the legal compliance of licenses in the mining, forestry, and palm oil sectors.

5 Prior to 2014, authority for IUP mining companies was held by district level governments (apart from authority over CCoW held by central government), between district energy and mining agency and district environmental agencies. In October 2014, this authority shifted to be divided across government agencies, particularly between the provincial energy and mining agency (ESDM) and the provincial environmental agency (DLH).
