# Supplementary material for: Patterns of infringement, risk, and impact driven by coal mining permits in Indonesia
Source: Ambio. 2023 Oct 27;53(2):242–56. doi: 10.1007/s13280-023-01944-y (PMC10774476; doi:10.1007/s13280-023-01944-y)
Supplement: Supplementary file 1 — Supplementary file1 (PDF 1740 kb) [file 13280_2023_1944_MOESM1_ESM.pdf]

Ambio

Supplementary information

Title: **Patterns of infringement, risk and impact driven by coal mining permits in Indonesia**

Authors

Tim T. Werner[1*], Tessa Toumbourou[1], Victor Maus[2,3], Martin C. Lukas[4], Laura J. Sonter[5,6], Muhamad Muhdar[7], Rebecca K. Runting[1], Anthony Bebbington[8]

Affiliations

[1] School of Geography, Earth and Atmospheric Sciences, University of Melbourne, 221 Bouverie Street, Carlton, VIC, Australia.

[2] Institute for Ecological Economics, Vienna University of economics and Business (WU), Vienna, Austria.

[3] Advancing Systems Analysis Program, International Institute for Applied Systems Analysis (IIASA), Laxenburg, Austria,

[4] Norwegian University of Science and Technology (NTNU), Department of Geography, 7491 Trondheim, Norway

[5] School of Earth and Environmental Sciences, The University of Queensland, St Lucia 4072, Queensland, Australia

[6] Centre for Biodiversity and Conservation Science, The University of Queensland, St Lucia 4072, Queensland, Australia

[7] Faculty of Law, Universitas Mulawarman, Jalan Sambaliung no. 1, Samarinda 75119, Indonesia

[8] Graduate School of Geography, Clark University, 950 Main St, Worcester, MA 01610, USA

[*] Corresponding author: tim.werner@unimelb.edu.au

Contents:

Summary of Figures 3

Summary of Tables 3

S1. History and description of coal mining governance in East Kalimantan 4

S2. Data collection and pre-processing methods 13

S3. Spatial overlay analysis methods and additional uncertainties 15

S4. Summary of data sources 16

S4.1 Water bodies and land clearing 16

S4.2 Concession / Mine permit data 20

S4.3 Analysis of mining areas 22

S4.4 Human Settlements 23

S5. Additional figures, tables, and detailed results 24

Supplementary References 39

Summary of Figures

Figure S1: Indonesia’s past coal production and regulatory milestones, 1981-2020.

Figure S2: Effective starting date of coal mining permits listed under the EDSM, PWYP and Auriga concession datasets in East Kalimantan (see Table S2).

Figure S3: Water transitions data derived from Pekel et al. (2016), indicating the emergence of water bodies over coal mining areas in southern parts of Samarinda.

Figure S4: Datasets WBA (Pekel et al., 2016, black and pink outlines) and WBB – shown in blue.

Figure S5: Landsat imagery composites assembled for each time period via Google Earth Engine.

Figure S6: Visualisation of operating coal permit areas obtained from three separate sources.

Fig S7: (a) Village point locations relative to proximity to coal pit water bodies; (b) Human settlement (raster or polygon) locations and spread relative to proximity to coal pit water bodies; (c) Coal pit water bodies emerged in operating coal concessions (south Samarinda area).

Figure S8: Overlay (1) - Village point locations with respect to their distance to pit ponds in operating coal concessions.

Figure S9: Overlay (2) – Village / settlement point data in relation to coal concessions, disaggregated by the operating status of the concessions.

Figure S10: Increase in the number of concessions (established by the effective date listed in the C-C Concession dataset) versus the increase in drownings over the same time period 2008-2019.

Figure S11: Overlap of the extended Nusantara capital region against coal exploration areas, and existing palm oil concessions.

Summary of Tables

Table S1: Types of coal mining permits in Indonesia

Table S2: Summary of spatial data on water bodies and land clearing

Table S3: Summary of spatial data on minutes permits / concession areas

Table S4: Summary of spatial data on coal mine areas (MA)

Table S5: Summary of spatial data on villages as point locations (VP) and urban areas as polygons or rasters (UA)

Table S6: Overlay (5) – Intersections between urban / settlement area polygons and mine permit areas (VA v C).

Table S7: Overlay (11) – Intersections between mine areas and coal concession (permit) areas (MA v C).

Table S8: Compiled records of victims of non-remediated mine pits in East Kalimantan.

S1. History and description of coal mining governance in East Kalimantan

Coal mining in East Kalimantan was first established under Dutch colonial administration, but intensified in the mid-1960s with the New Order regime's emphasis on foreign investment. Two key laws introduced in 1967 stimulated industrial-scale coal mining. The first was Law 1/1967 on Foreign Investment which granted foreign mining investors generous tax concessions, profit repatriation and 30-year contractual terms (Robinson, 2016). The second was Law 11/1967 on Basic Regulation of Mining, which established a Contract of Work system, setting in place provisions favourable to mining companies to attract foreign investors. Under this law, foreign mining investors and operators enter a formal agreement with the Indonesian government to undertake mining on the government's behalf (Butt and Lindsey, 2018). In 1981, a Presidential Instruction (no. 49/1981)[1] enacted the initial contractual arrangements for coal mining contractors to operate, beginning industrial scale coal mining in Indonesia (Daulay, 1994). The first bilateral contracts for large-scale mines – known as Coal Contracts of Work (CCoW) – signed by the government with foreign coal mining companies, known as first generation CCoW, were exempt from having to adhere to any changes to Indonesian law that occur after the contract is signed (OCallaghan, 2010). CCoWs were not required to plan for mine closure and reclamation (Robinson, 2016). The first generation CCoWs, signed between 1981 and 1990, continue to produce the majority of Indonesia's coal; in 2015, they accounted for over 50% of Indonesia's total coal output (Friederich and van Leeuwen, 2017). The majority (11 out of 12) were located in East Kalimantan – the other in West Sumatra. There have been three subsequent phases of CCoW, as of 2018 there were 74 CCoW still in operation, making up the majority (61% in 2016) of coal production (Atteridge et al., 2018).

1999-2009: regional autonomy: In 1998, at the end of the New Order and the beginning of Indonesia's Reformation (*Reformasi*), a political transition towards a more open and democratic system resulted in the redistribution of power to regional districts. In 1999, Law 22 on Regional Government and Law 25 on Fiscal Balancing between the Central and Regional Governments were enacted, formalised the devolution of authority over natural resources to district and municipal level governments (Butt and Lindsey, 2018; Fox et al., 2005). Coming into

[1] Presidential Decree no. 49 of 1981 concerning Main Provisions of Coal Contract of Work between State Coal Mining Company and Private Contractor.

operation in 2001, an implementing regulation (Government Regulation no. 75/2001) enacted decentralisation in the mining sector, granting district heads, mayors, governors and ministers the authority to issue coal contracts according to their respective jurisdictions and authorities (Devi, 2013; Velentina, 2020). Legislation around mining was vague and poorly drafted, allowing sub-national authorities to derive revenues from the exploitation of natural resources, resulting in a proliferation of mining permits (Fox et al., 2005). In 2004, laws enacted direct elections for heads of local subnational government, including for the role of district head (Erb et al., 2021; Mietzner, 2007). The first direct elections of district heads in 2005 'generated a rise in the cost of politics' (particularly for campaigns and the increasing use of money politics), opening up opportunities for mining companies to offer funds in return for mining permits (Erb et al., 2021, p. 91). The notable increase in mining operations in 2006, indicated in Figure S2, is likely linked to this phenomenon; after taking office, elected officials issued permits they had promised to mining companies who had bankrolled their campaigns.

As evidenced in Figure S1. below of coal production in Indonesia, between 2000 to 2009, a massive and loosely regulated expansion of coal mining ensued such that Indonesia would move from being a marginal to major player in the global production of coal, with production increasing ~460% in this time. By 2005, Indonesia had become the largest exporter of thermal coal in the world (Atteridge et al., 2018, Dudley, 2019)Indonesia has since maintained that position; at a peak period of coal production in 2013, Indonesia supplied 38% of global steam coal exports (Friederich and van Leeuwen, 2017).

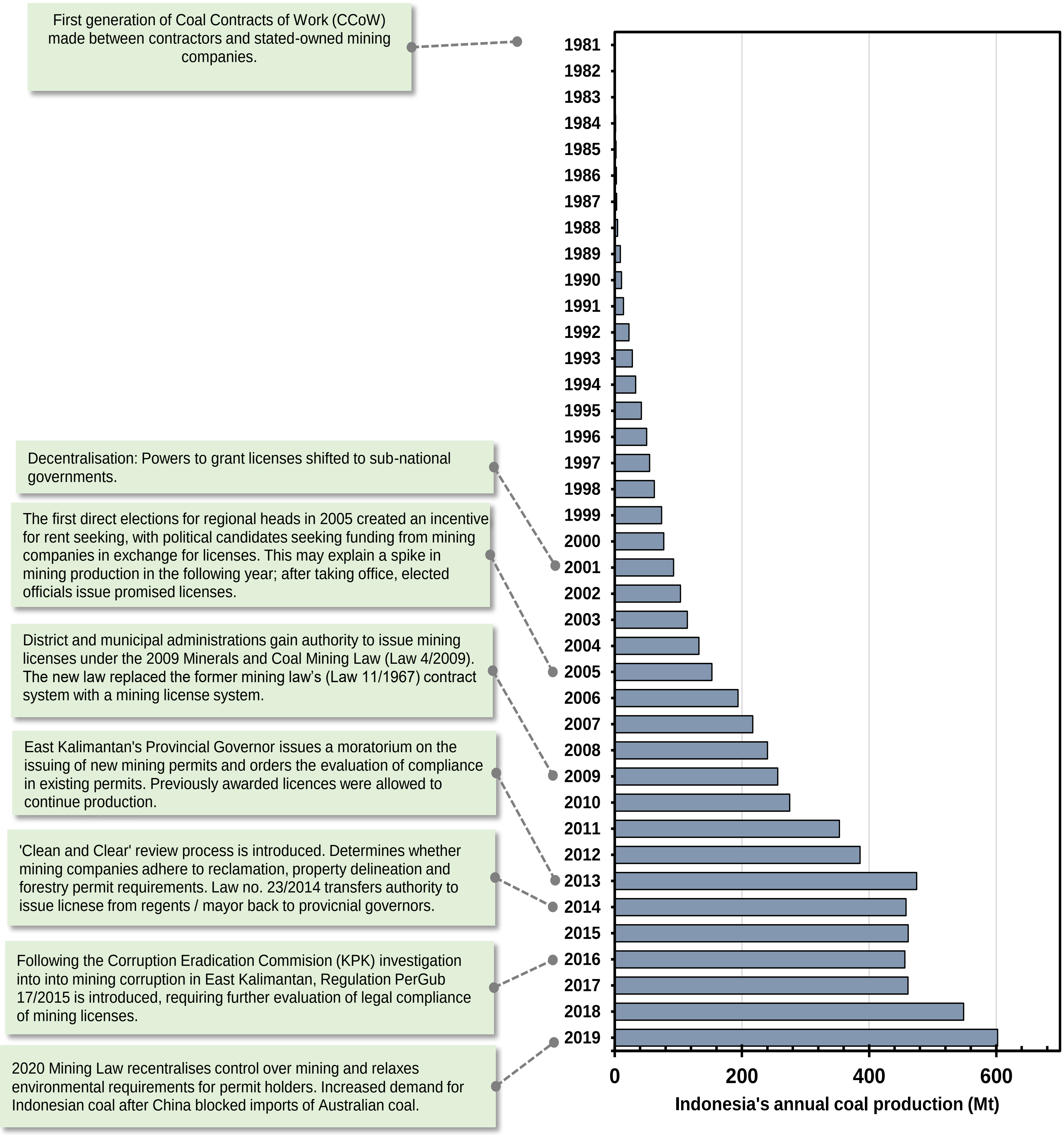

Figure S1: Indonesia's past coal production and regulatory milestones, 1981-2020 (Adapted from: Dudley (2019), Atteridge et al. (2018), Toumbourou et al. (2020)). Note that these data likely do not encompass the production/shipping of coal derived from illegal or otherwise undocumented sources.

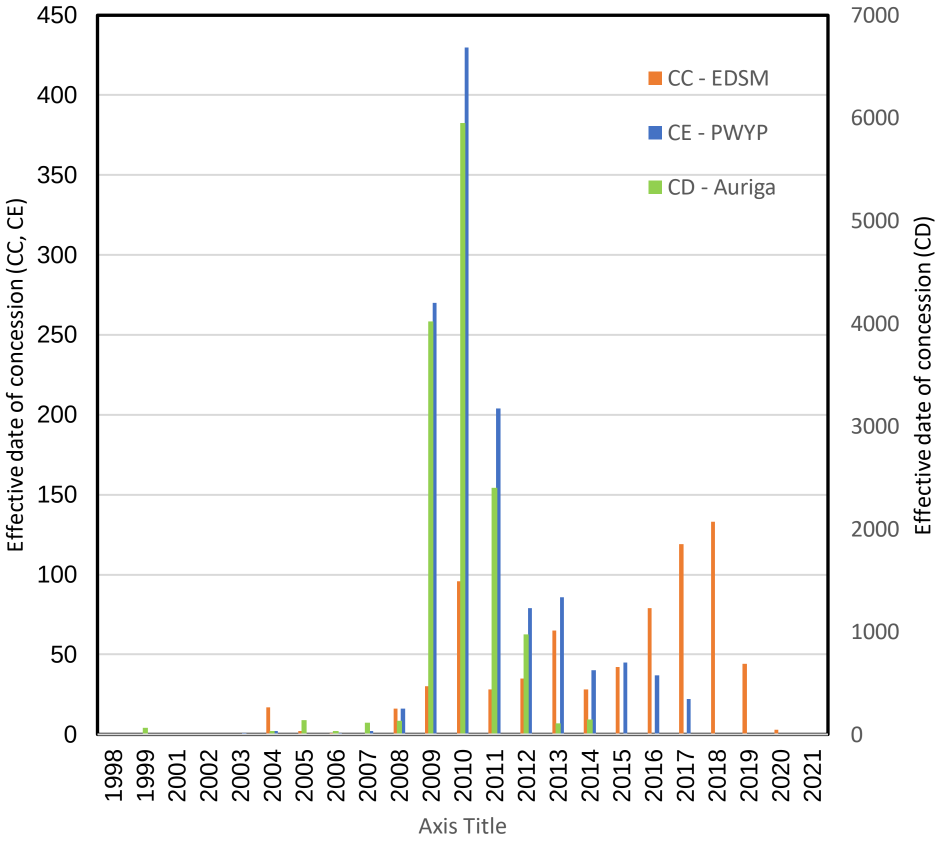

Figure S2: Effective starting date of coal mining permits listed under the EDSM, PWYP and Auriga concession datasets in East Kalimantan (see Table S2).

2009 to 2020: predominance of small to medium mining permits: In 2009 the Contract of Work system was replaced with a new system, with the issuance of a new Law on Mineral and Coal Mining (Law 4/2009). Under this new mining system, mining business permits (known as *Izin Usaha Pertambangan*, or IUP) were introduced as requirements for coal exploration and production, replacing the contract system. CCoW issued under the former contract system were able to continue to operate until their expiry, at which time they would be transferred to a new permit system, requiring that miners obtain an IUP (Atteridge et al., 2018). Under the Mining Law No. 4/2009, this transfer was required to be conducted through an auction to offer these contracts to state-owned companies first. Coal production increased in this period, spiking in 2011 correlating to a peak in coal prices and strong demand from India, then slumping between

late-2013 to 2016 due to a slackening of prices and reduced export demand (Friederich and van Leeuwen, 2017).[2,3]

Alongside fluctuations in prices – driven by changing external demand – domestic regulatory factors introduced over this period may have also affected Indonesia's coal production. In 2010, a regulation on reclamation and mine closure was introduced (Government Regulation No. 78/2010 on Reclamation and Mine Closure), requiring that a reclamation and post-mining activities plan is submitted in order to obtain an operation mining permit. A IUP holder must also set aside Reclamation Activities Guarantee (*jaminan reklamasi*) in the form of a time deposit in a joint account with an Indonesian government owned bank. The implementation of the Public Information Disclosure Law (Law no. 14 of 2008) in 2010, gave civil society new rights to access previously inaccessible public information. Environment groups in EK leveraged their new rights to request mining permit documents for mines issued in the province, revealing that 71% of Samarinda, EK's capital city, had been allocated permits for coal mining. These figures were used in media campaigns to raise awareness of the extent of mining permit allocations, and informed civil society strategies, from lobbying, litigation to advising on policy formulation (Toumbourou et al., 2020).

In early 2014, the Corruption Eradication Commission (*Komisi Pemberantasan Korupsi*, KPK) initiated a scheme to investigate illegalities in the mining sector in Indonesia. Beginning in February 2014, the investigations systematically evaluated permits in 12 major coal producing provinces across Indonesia, including East Kalimantan. Within a year, investigations had identified that 40% of permits were in violation of permit and environmental regulations. In October 2014, the central government issued Regional Governance Law (no. 23 of 2014) that moved authority over mining concessions to provincial and central governments, depending on concession size. This recentralisation of authority over mining coincided with a temporary downturn in global coal prices (driven also by reduced demand from China, Indonesia's major coal importer), which led to a reduction of Indonesia's coal exports (Funfgeld, 2018).

---

[2] https://www.reuters.com/article/coal-indonesia-idUSJKB00404020101014

[3] https://www.thejakartapost.com/news/2016/02/18/kalimantan-sumatra-worst-hit-cheap-commodities.html

Text box: *Clean and Clear certification*

To address overlaps and other inconsistencies in the mining sector, on 12 September 2012 the Minister of Energy and Mineral Resources issued a procedure for determining whether coal licences complied with mining regulations, known as clean and clear (see Velentina, 2020). Following several amendments, the procedure for determining clean and clear was formalised on 30 December 2015 with an MoEMR regulation (Ministerial Regulation No. 43/2015 regarding Procedures to Evaluate the Issuance of Mining Business Licences). The Ministerial Regulation sets out an audit process for determining whether companies meet minimal legal requirements relevant to permit holders.[4] Conducting reclamation however was not a criteria of clean and clear certification. The Clean and Clear certification system was a desktop exercise to ensure that all required documentation is in place, and does not require a site assessment – hence it does not reflect how practices adhere to environmental and human health protection laws. Governors across Indonesia were given until 31 March 2017 to ensure that mining permits met clean and clear standards. In cases where a review has determined non-compliance, a governor or the MoEMR has the authority to revoke or amend the IUP licence. IUP that are compliant with the regulations achieve a 'Clean and Clear' (CnC) certificate, and were announced on a public list of Clean and Clear status IUP. Observers have pointed out that the sanction of revoking the license of a non-Clean and Clear permit holder is a perverse policy, as this then transfer the burden of cost of resolving 'illegalities' with these mining companies' operations to the state. Funds set aside in the form of a Reclamation Guarantee deposit are supposed to cover the costs of reclamation and clean up, but interviewees observed that the real costs far exceed Reclamation Guarantee funds, when the funds are set aside.

In February 2018, the MoEMR issued a regulation (MoEMR regulation No. 11/2018 regarding Procedures for the Granting of Area, Licensing and Reporting on Mineral and Coal Mining Activities), which states that IUPs that have been issued with Clean and Clear shall

[4] The regulation sets five criteria for assessing permit documentations' compliance with relevant laws, including: (1) administrative – ensuring the mining process following regulations accordingly (including that an exploration permit was granted prior to an operation permit); (2) areal – having no property delineation issues (including no overlap with other land uses/license allocations over a concession area, and that the appropriate type of permit was granted for the area's zoning); (3) technical – that an exploration report was completed for IUP-E holders, or an exploration and feasibility study was conducted, for IUP-O holders; (4) environmental – conforming to any environmental commitments (including having conducted an environmental document, and that this has been approved by the relevant instance); (5) financial – complying with all tax and non-tax financial obligations (including evidence of payment of royalties).

remain valid, but IUPs issued after the enactment of this regulation no longer require Clean and Clear status.[5]

Since this time, Indonesia's major recent energy and economic development plans have focused on increasing coal-fired electricity capacity nationally, driving an increase in domestic coal consumption to replace diminishing export demand. The National Energy Policy of 2014 set a target of minimising oil consumption and in replacement increasing coal to supply national energy needs (OECD, 2019). The medium-term development plan for 2015-2019 aimed to set a cap on coal production, from 425 Mt/y in 2015 to 400 Mt/y in 2019 in order to protect supply for national electricity production (Nugroho, 2019). These caps were however exceeded, indicating that the state has limited control over coal production (IEA, 2020). Coal production spiked significantly in 2018 (reaching 549 Mt) and again in 2019 (reaching 616 Mt). Indonesia's share of global coal production rose in this period, resisting the trend of many other countries looking to decarbonise their energy sectors. Indonesia remains a major coal exporter, second only to Australia in 2018 (Dudley, 2019). India and China constitute the major buyers, while a growing domestic market supplies Indonesia's electricity generation demands (Friederich and van Leeuwen, 2017).

2020: recentralisation of mining authority: In 2020, laws overseeing mining were revised again, with the issuance of Law on Mineral and Coal Mining (Law No. 4/2020). A major change with this law is the recentralisation of authority over mining, which has moved to the central government. The revised law further relaxes mining regulations, and extends CCoW for two consecutive ten-year periods (a total of twenty years) without an auction to offer these contracts to state-owned companies first, as was required in the former version of the law. The changes also preserve the lack of clarity around what is meant by mine rehabilitation in the previous law, that has allowed companies to avoid refilling mining pits. The new law removes limitations on the size of a mining concession allowed for a single IUP.

---

[5] https://kemlu.go.id/download/L1NoYXJlZCUyMERvY3VtZW50cy9NSU5JTkclMjBJTiUyMElORE9ORVNJQSUyMC0lMjBJTlZFU1RBVElPTiUyMEFORCUyMFRBWEFUSU9OJTIwR1VJREUlMjAyMDE5LnBkZg== ; https://bisnis.tempo.co/read/1060068/esdm-cabut-regulasi-penataan-izin-tambang/full&view=ok

Another change affecting governance of the mining sector is with the Law on Job Creation (Law No. 11/2020 or Omnibus Law), which came into effect on 5th November 2020.[6] The Law No. 11/2020 introduced a total of 79 legal amendments and 51 implementing regulations, with the purpose of simplifying rules and administrative systems to increase foreign investment. The legal changes remove the current prerequisite for an environmental impact assessment (called AMDAL) prior to establishing any mine or other development, instead only requiring activities deemed 'high risk' to conduct an AMDAL, with activities deemed low risk only requiring to be registered, and moderate risk to conduct an 'environmental feasibility study'. Criminal charges for violations of environmental regulations are removed, replaced only with administrative sanctions. The changes also remove the requirement for involvement of environmental experts or other civil society observers in the AMDAL or environmental feasibility study, an important measure to ensure transparency and rigour.

A summary of various permit types provided in Indonesian law is provided in Table S1.

International regulatory environment: There are a range of international conventions that potentially play a role in the governance of mining, e.g., the International Labour Organisation Convention No. 176 that sets standards for health and safety around mine sites, promoting safe working conditions for miners, or the World Heritage Convention, or indeed the Paris Agreement. These, however, are largely not explicitly directed at mining, and are largely voluntary or otherwise non-binding. Other international standards or organisations, such as the International Council on Mining and Metals (ICMM), the Global Reporting initiative (GRI), Extractive Industries Transparency Initiative (EITI), set international benchmarks for mining operation, governance, and reporting.

Each of these conventions and organisations have some influence in Indonesia, although their specific role in coal mining in East Kalimantan has been limited, given that even the most explicit local regulations are not routinely adhered to.

[6] https://www.thejakartapost.com/paper/2020/10/15/jobs-law-unlikely-to-boost-mining.html

Table S1: Types of coal mining permits in Indonesia

Licence Type	Description
PKP2B (Coal Contract of Work, CCoW / *Perjanjian Karya Pengusahaan Pertambangan Batu Bara*)	CCoW were granted by the MoEMR under the pre-2009 mining system (in accordance with Law 11/1967). CCoW are no longer granted, but remain in place until they expire, at which time they may be replaced in the form of IUP or IUPK. Generally, CCoW have been for larger operations, whereby concessions size was arranged through contractual negotiation between the central government and a company. No changes to coal contracts can be made without the agreement of both parties. CCoW are not required to obtain clean and clear status. Under the 2009 Mining Laws CCoW remain in place until their expiry, at which point they would be transferred to the new permit system.
IUP (Mining Business Licence / *Izin Usaha Pertambangan*)[7]	An IUP is a general license granted to conduct mining in areas designed by the government as designated mining areas. Under the 2009 Mining Law system, authority for issuing IUP was with district heads or governors, depending on the size and location of a mine. Law No. 23/2014 moved authority for issuing IUP to provincial governments. Licenses were issued by a governor or the MoEMR, depending on the size and location of the mine. Law No. 4/2020 moved authority for issuing IUP to the central government.<br><br>IUP are granted in two phases. First, an exploration license (*IUP-explorasi*) is granted for surveying and feasibility studies. This is then followed with a production operation license (*IUP-Operasi Produksi*) granted for construction, operations, processing, transportation, and selling activities. Mining license holders do not own their concession area, and have no automatic rights to exploit other minerals. Concession size is capped at 15,000 ha.[8]
IUPK (Special Mining Business Licence / *Izin Usaha Pertambangan Khusus*)	An IUPK is a license granted to conduct mining in specific state reserve areas for national strategic interests. An IUPK is commonly granted as a continuation of CCoW. State- and region-owned entities have priority in obtaining this kind of licence. Like the IUP, IUPK are granted in two phases: exploration and production operations. Concession size is capped at 15,000 ha.
IPR (People's Mining License / *Izin Pertambangan Rakyat*)	An IPR is a license for conducting mining in an area of limited size for small scale mining. IPR can only be granted to an individual Indonesian investors, up to a maximum of one hectare, to a community group (max. 5 hectares), or to a cooperative (max. 10 hectares)

Source: Atteridge (2018); Devi (2013); PwC Indonesia (2019)

[7] Legislation limits mining to areas designated in the national spatial plan to have potential mineral or coal reserves (*wilayah pertambangan*). Once categorized, an area can be designated a mining permit area (*wilayah izin usaha pertambangan - WIUP*). A IUP can only be issued to a mining company for areas that have been designated WIUP.

[8] An IUJP (Mining Services Business Licence / *Izin Usaha Jasa Pertambangan*) is a mining business license granted for performing core mining service business activities in relation to certain phases/parts of the mining business activities. This must be obtained by a company wishing to provide services to an IUP/IUPK holder.

S2. Data collection and pre-processing methods

We assessed satellite imagery and spatial data obtained from government, community, and media sources. We used Google Earth Engine, wherein JavaScript code was written to obtain and pre-process cloud-free satellite imagery for the boundary of East Kalimantan. Composites of Landsat (7, 8) surface reflectance imagery from three periods were created and clipped to the boundary of the province. Period A (2005-01-01 to 2009-12-31) encompasses geomedian land cover (see Phan et al., 2020) leading up to and including the earliest stages of the 2009 Mining law that saw large-scale license granting across the province. It therefore represents a baseline image composite. Period B (2010-01-01 to 2017-12-31) represents a period in which mining in the province underwent significant expansion, and during which most drownings took place. Period C (2018-01-01 to 2020-03-21) represents the recent land cover, up until the date of the most recent recorded drowning at the time of assessment. Given the region's humid tropical climate, it was necessary to assess such periods extending multiple years to produce a clean, province-wide composite image without cloud cover. A reducer was used to remove clouds (high value) and cloud shadows (low value). Despite these measures, some pixels from Period C were consistently cloud-covered across the entire period used, in which case a cloud bit mask was used to filter out remaining cloud and cloud shadow pixels from this imagery. Period C was then mosaiced with Period B to produce the most recent possible cloud free composite of the province. These visualisations are shown in supplementary Fig. S5.

Coal mine concession data are not openly available in Indonesia; however, these data were obtained through contact with Indonesian government departments, systematic review of online spatial data portals, and contact with Indonesian NGOs that had sourced data through freedom of information mechanisms. Although the 2010 Freedom of Information Act gives Indonesian citizens the right to access public data on public domain issues, obtaining data from government remains notoriously difficult. Civil society organisations and researchers are often endlessly sent back and forth

(‘pingponged’) between persons and agencies. Letters of request are rarely responded to within the period set out in the law, and data provided is often incomplete or poor. We ultimately sourced three distinct spatial datasets of permits (concessions), highlighting slight differences (see Fig S6). These differences may be due to ongoing changes in the number and location of permit areas between the times at which the different datasets were compiled, although inconsistent record keeping is also a known issue in East Kalimantan, whereby different agencies with different responsibilities in relation to land or environmental management maintain different records. All concession datasets were ostensibly recent (to the time of manuscript preparation), having listed permits with recent effective dates, meaning each dataset was considered equally valid. These spatial datasets were also supported by two spreadsheet records of coal mine permits obtained via contact with NGOs in Indonesia (supplementary Table S2).

Areas of active mining operations were determined from Maus et al. (2020a, 2020b), as well as government land cover maps maintained by the Ministry of Environment and Forestry (MoEF) and originally developed by methods described by Wijaya et al. (2015), validated by Kiswanto et al. (2018), and updated in Sonter et al. (2020b). Such maps were also used to validate assessments of water body areas, human settlements and cleared land. Water bodies were also identified through support vector machine learning supervised classification of the Google Earth Engine composites (described further below), and surface water transition data from Pekel et al. (2016).

Village location data were obtained through open-source online settlement sources, as well as through updating and validation of a village dataset from The Nature Conservancy. Additional village location datasets were obtained as points via Open Street Maps and Populated Places datasets available through the ArcGIS Online spatial data portal. Human settlement areas (polygons and rasters) were identified from The Global Human Settlement built-up area grid, derived from Sentinel-1 data (Florczyk, 2019), the Global Urban Footprint datasets (Esch et al., 2014), land cover data from Kiswanto et al. (2018), and the MoEF land cover maps.

S3. Spatial overlay analysis methods and additional uncertainties

All spatial data were imported into ArcGIS Pro for visualisation, data management, and analysis. All imagery, point, polygon, and raster datasets were geometrically referenced using the Universal Transverse Mercator (UTM) projection system, part of the World Geodetic System (WGS) 1984. Spatial relationships between the datasets were analysed using the intersection and proximity tools as per Fig. 2, with multiple spatial overlays conducted for each individual dataset. Output summaries of the extent to which the various features overlapped, or the distribution of proximities were tabulated in Microsoft Excel and summarised in the following sections.

Additional uncertainty arose in the identification of fatalities. To determine the locations, we used the name of the operating company reported in news media to identify the correct concession, and then we selected a pit within that concession, based on the best information available in public media reports. For drowning locations classified as “Medium” or “Low” accuracy (see Table S8), it is possible that the drowning actually took place outside the concession, while the location of the drowning was marked as happening within the concession. This does not imply that the drowning was not the result of that particular company’s operations (as news reports typically stated the company operating the hazardous pit), but simply that a different individual pit other than the one pinpointed in the analysis may have been the site of the death.

S4. Summary of data sources

S4.1 Water bodies and land clearing

Table S2: Summary of spatial data on water bodies and land clearing

Dataset	Date relevant	Source	Description / Notes
**Water bodies (WB)**			
WBA - Global Surface Water 1984-2020	1984-2020	Pekel et al. (2016) - Jean-Francois Pekel, Andrew Cottam, Noel Gorelick, Alan S. Belward, High-resolution mapping of global surface water and its long-term changes. Nature 540, 418-422 (2016). Data downloaded via <https://global-surface-water.appspot.com/download>,	This dataset contains information on the area of surface water determined from Copernicus satellite data, displayed as raster data at 25 m resolution. Contains data on surface water occurrence, transitions, seasonality, recurrence, and change intensity. This dataset was used to identify which water bodies had emerged in coal permit area boundaries, and since those permits were granted. This dataset was validated against the google earth image composites described in dataset WBB, finding high spatial consistency via visual inspection. See Figures S3 and S4.
WBB - Google Earth Landsat Image Composite Water Cover	2020	Google Earth Engine, sourcing Landsat 8 Composite cloud-free imagery for the period 2017-2020.	Support vector machine learning of the Google Earth Engine Exported composites allowed for surface water bodies as of 2017-2020 to be identified. As shown in Fig S4, there was strong agreement between the WBA and WBB datasets, supporting our later classifications of land cover, and supporting the use of WBA for assessing coal-associated water bodies.
**Land clearing (LC) and additional land cover data considered.**			
LCA – Global Forest Change (Hansen et al., 2013)	2000-2020	Hansen, M. C., P. V. Potapov, R. Moore, M. Hancher, S. A. Turubanova, A. Tyukavina, D. Thau, S. V. Stehman, S. J. Goetz, T. R. Loveland, A. Kommareddy, A. Egorov, L. Chini, C. O. Justice, and J. R. G. Townshend. 2013. “High-Resolution Global Maps of 21st-Century Forest Cover Change.” Science 342 (15 November): 850–53.	Documents tree cover and loss annually from 2000-2019, updated from the original 2013 publication. Tree cover represents vegetation taller than 5m height per <https://glad.earthengine.app/view/global-forest-change#dl=1;old=off;bl=off;lon=20;lat=10;zoom=3>. Accessed March 2020.
LCB – Landsat Image Processing	2005-2020	Landsat 7 and Landsat 8 Imagery compiled via Google Earth Engine, explained in main text section: ‘Remote sensing image classification and uncertainty assessment’.	Compiled into three composite periods displayed in natural colour and false colour in Figure S5 below.
Land cover polygons 2015-2040	2017-2040	Sonter, L.J., Simmonds, J.S., Watson, J.E., Jones, J.P., Kiesecker, J.M., Costa, H.M., Bennun, L., Edwards, S., Grantham, H.S., Griffiths, V.F. (2020) Local conditions and policy design determine whether ecological compensation can achieve No Net Loss goals. Nature Communications 11, 1-11.	Sonter et al. modelled potential future land use changes in this province considering multiple land cover classes. These were used to consider the potential extent of mine areas up to 2040 in East Kalimantan.
Land cover polygons	2013-2017	Wijaya et al. (2015) and Indonesian Ministry of Energy and Mineral Resources	Land cover data for east Kalimantan, updated and republished on the Ministry of Energy and Mineral Resources website.
Oil Palm concessions	ca. 2015	Obtained from the Global Forest Watch, via ArcGIS Pro Online Data Portal. Cites Indonesia Ministry of Forestry.	Downloaded 3 April 2020. Contains polygons of oil palm concessions per Global Forest Watch ca 2015, overlaying extensively with coal concessions. Interviews conducted in past work indicate that palm oil concessions and coal concessions are a demonstration of one form of extraction enabling another.

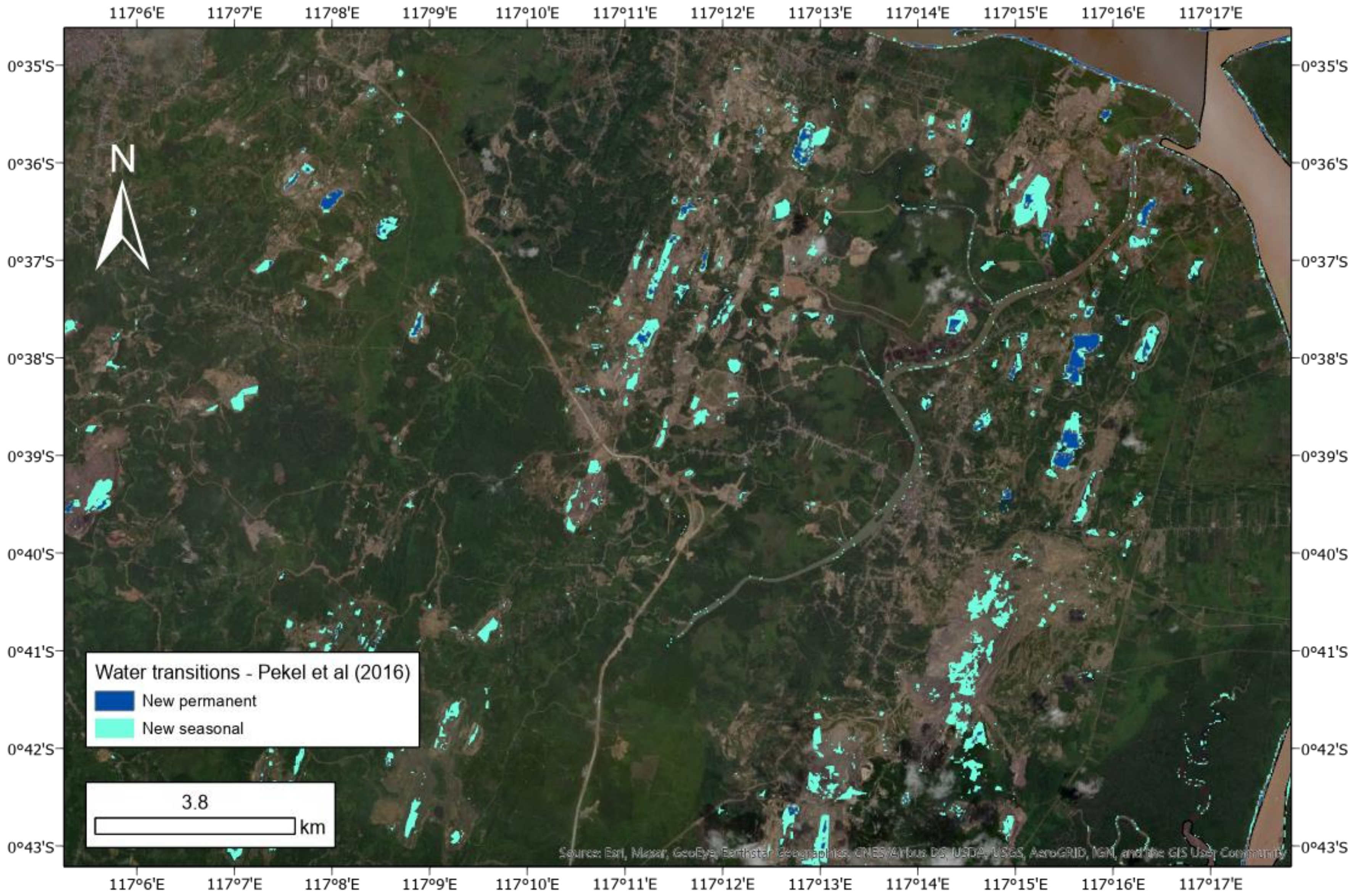

Figure S3: Water transitions data derived from Pekel et al. (2016), indicating the emergence of water bodies over coal mining areas in southern parts of Samarinda.

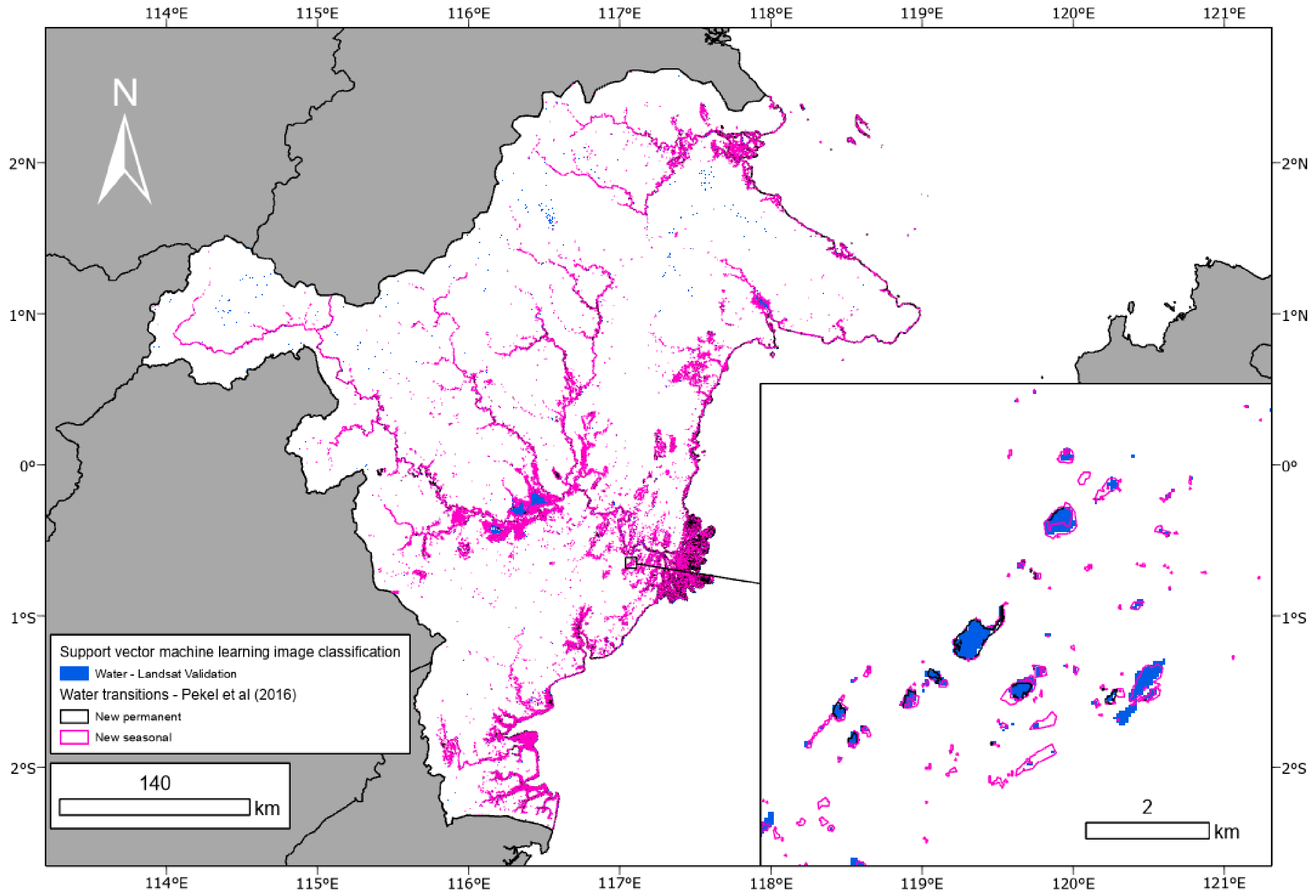

Figure S4: Datasets WBA (Pekel et al., 2016, black and pink outlines) and WBB – shown in blue. Agreement between these datasets supported the validation of subsequent landcover classifications and the use of Pekel et al. (2016) data for assessing mine-related water hazards.

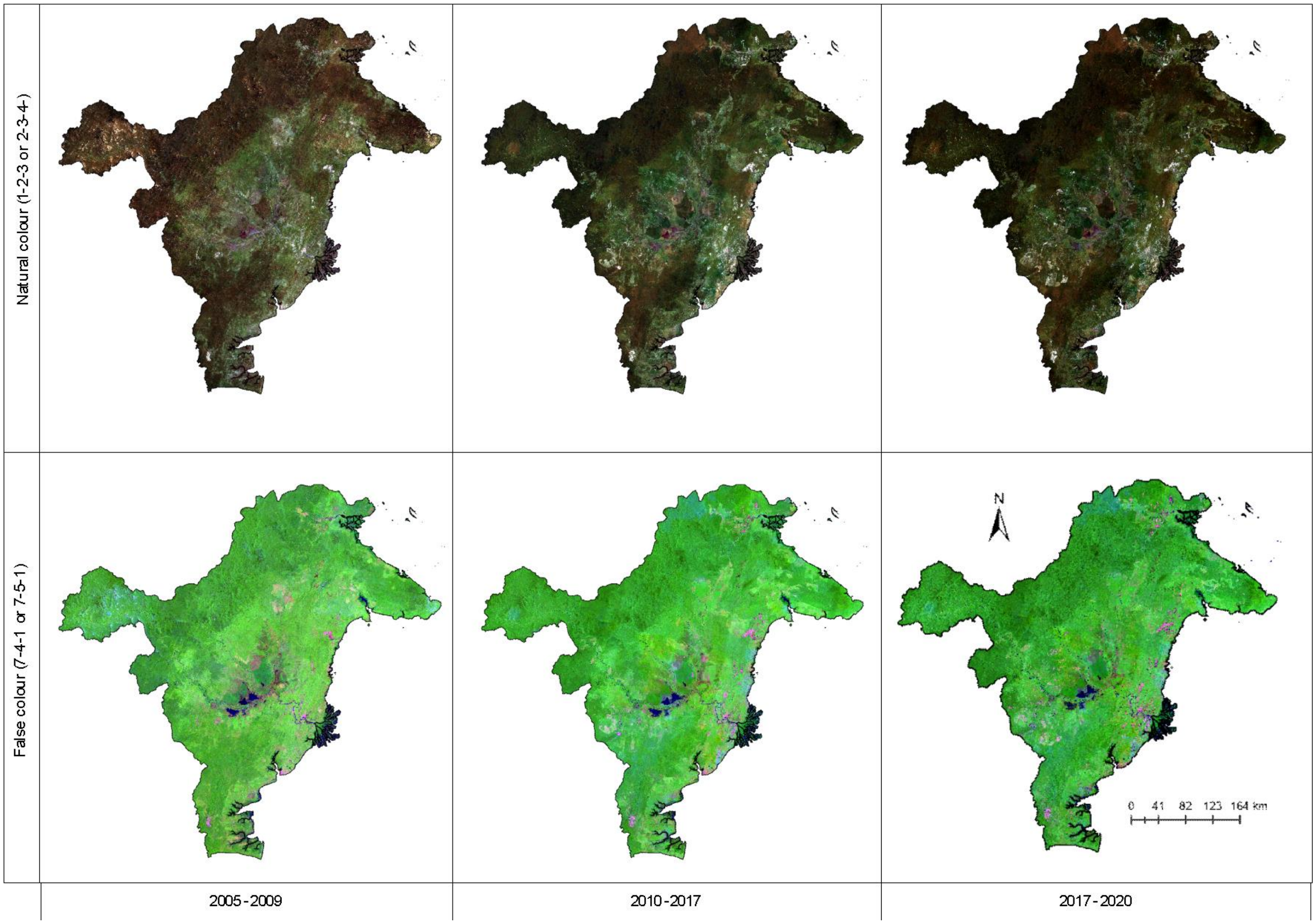

Figure S5: Landsat imagery composites assembled for each time period via Google Earth Engine, displayed in natural colours (bands 1-2-3 for Landsat 7 and 2-3-4 for Landsat 8) in false-colours to highlight mine areas (7-4-1 for Landsat 7 and 7-5-1 for Landsat 8).

S4.2 Concession / Mine permit data

Table S3: Summary of spatial data on minutes permits / concession areas

ID	Date relevant	Source	Data sorted by Batubara [Coal] and Kalimantan Timur [East Kalimantan]. N; Category total area (% of all total area)				Notes / Data features
			IUP	PKP2B (CCoW)	Other / not classified	N (Total)	
C-A: JATAM (WIUP_EDSM_Kaltim)	Ca. 2016	Obtained via direct contact from JATAM.	N=476; 1,253,913 ha (62%)<br>Exploration: N=61; 233,370.82 ha (11.5%)<br>Operating Production: N= 385, 1,020,452.14 ha (50%)	N=39; 766,971 ha 37%<br>Exploration: N=5; 148,335.16 ha (7.3%)<br>Operating Production: N=34; 618,635 ha (30.6%)	-	N=485; 2,020,883.92 ha (100%)<br>Exploration: N=66; 381,706 ha (18.9%)<br>Operating Production:N=419; 1,639,177.9 ha (81.1%)	Inputting of coordinates covered in the SK (decree) license document issued by the municipal/district government. East Kalimantan energy and mineral resources agency (ESDM) via JATAM East Kalimantan. Crosschecked with the map attached to the East Kalimantan spatial plan 2016-2036 (Perda = provincial regulation formalising the spatial plan).
C-B: The Nature Conservancy (EastKal_CoalMining_Update_UTM5)	Ca. 2018	Obtained via ArcGIS Online: <https://www.arcgis.com/home/item.html?id=a75330d4bd0b49e6ac4bb84e56e44f32><br><br>Uploaded via The Nature Conservancy Indonesia	-	-	See right.	N=12,101; 3,963,116.1 ha (100%)<br>Exploration: N=2,671; 1,680,681 ha (42.4%)<br>Exploitation: N=37; 1153.4 ha (0.03%)<br>Operating Production: N=6,905; 1,359,712.6 ha (34.3%)<br>Pencadangan: ("Backup"): N=41; 7,288.9 ha (0.18%)<br>Penyelidikan Um* ("Under investigation": N=4; 7,810 ha (0.19%)<br>Not classified: N= 2443; 906,470.1 ha (22.9%)	This dataset covers the largest spatial area among all the concession data obtained (see Figure S6). A significant portion of these data are vaguely classified (e.g. as under investigation or backup) which may explain the increased total coverage. The areas visualised in Figure S6 depict operating production areas only. Only 12% (N= 1453) of sites within this dataset are classified according to the IUP/PKP2B (CCoW) status and hence only total areas and operating status are summarised here.
C-C: EDSM / Indonesian Ministry of Energy and Mineral Resources	Ca. 2020	Obtained via ArcGIS Online: citing ESDM (Indonesian Ministry of Energy and Mineral Resources. See also: <https://momi.minerba.esdm.go.id/public/>	N=608; 1,027,944.8 ha (56.1%)<br>Exploration: N=4; 4777.5 ha (0.3%)<br>Operating Production: N= 604; 1,023,167.2 ha (55.8%)	N=131; 804,214.6 ha (43.9%)<br>Exploration: N=4, 74,195.7 ha (4%)<br>Operating Production: N=127; 730,018.9 (39.8%)	-	N=739; 1,832,159.3 ha (100%)<br>Exploration: N=8; 78,973.2 ha (4.3%)<br>Operating Production: N= 731; 1,753,186. 1 ha (95.7%)	This data is publicly viewable (albeit not downloadable) on an official Indonesian government website. The magnitude of areas in each category is roughly similar to that of dataset CA. These datasets are considered to best represent currently active sites, whereas datasets C-B, C-D and C-E are considered to best represent cumulative permit areas granted over time.
C-D: (via public information request of Auriga)	Ca. 2015-2016	Obtained via contact at Clark Univ. In turn obtained via Auriga and Jatam. Likely obtained via public information requests.	N=4769, 4,837,045.4 ha, 83.1%<br>Exploration: N=3409, 3,998,981.8 ha, 68.7%<br>Operating Production: N= 1360; 838,063.5 ha (14.4%)	N=442; 976,891.5 ha (16.8%)<br>Exploration: N=64; 263,924 ha (4.5%)<br>Operating Production: N=378; 712,967.5 ha (12.2%)	N=6, 9925.5 ha, 0.2%<br><br>(all Exploration)	N=5217; 5,823,862.377 ha (100%)<br>Exploration: N=3479; 4,272,831.3 ha (73.4%)<br>Operating Production: N= 1738; 1,551,031.1 ha (26.6%)	Notes the "generation" of the PKP2B licenses. (e.g. GEN I, GEN II…). While noted to be relevant up to ca. 2015-16, there are few new licenses granted since 2013 in this dataset, which is consistent with the moratorium. Note that this is not a spatial dataset- but rather a list of government records that were analysed for comparative purposes. It covers a large number of entries dating back to 1998.
C-E: (via public information request of Publish What You Pay)	Ca. 2017	Sourced via contact at Publish What You Pay Indonesia	-	-	-	N=1404; 3,565,318.27 ha (100%)<br>Exploration: N=775; 2,468,334.6 ha (69.2%)<br>Operating Production: N= 573; 927127.9 ha (26%)<br>Other (General investigation and Exploitation): N=49; 169855.8 ha (4.8%)	Presents data up to ca. 2017, however data from 1998 onward are included, leading to a larger number of entries. This dataset is also tabular only, although the size of the concessions is also reported, as with dataset C-D. It is noted that some indication of IUP status is given a column outlining the decision letter number, however it is not possible to summarise breakdown statistics according to the type of contract.

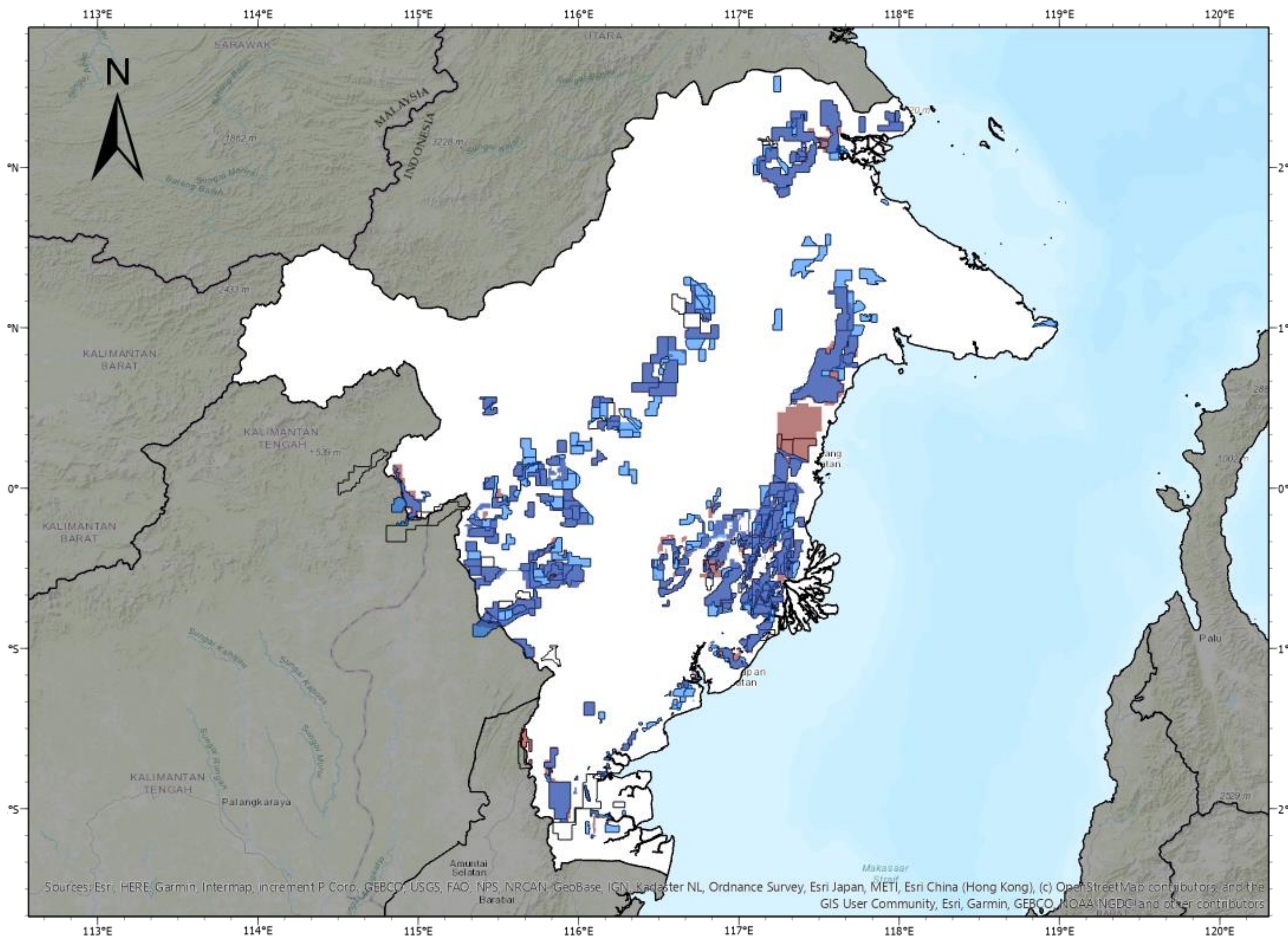

Figure S6: Visualisation of operating coal permit areas obtained from three separate sources. Blue areas represent concessions noted in the JATAM (C-A) dataset. These appear purple when overlain by the red areas (i.e. The Nature Conservancy, C-B dataset). Areas with black outlines represent the permit areas of the EDSM (C-C) dataset. Note that while there is overall agreement about which areas have been granted operating permits, these datasets are not entirely consistent, as some areas can been to be only blue, only red, or only outlined black, as well as a combination of only two of the three datasets. For the purposes of overlay analyses, some assessments were carried out on individual datasets, as well as the total combined areas of these datasets. In doing so, we constructed a picture of all potential operating coal sites, as well as perspectives of government- or NGO-recorded sites. Differences between these datasets reflect known issues with record keeping in East Kalimantan, but may also reflect slightly different snapshots in time, as coal concession areas become active and inactive over time, and may be removed from some lists.

S4.3 Analysis of mining areas

Table S4: Summary of spatial data on coal mine areas (MA)

ID	Data type	Date relevant	Source	Data Summary, Notes and Features.
**Mine Areas (MA)**				
M-A-A: Indonesian Government (2017)	Polygon	Ca. 2017	Land cover and land use change datasets derived from the Ministry of Environment and Forestry (KLHK 2018), based on Landsat interpretation described in Wijaya, A. et al. (2015). Assessment of large-scale land cover change classifications and drivers of deforestation in Indonesia. ISPRS - Int. Arch. Photogramm. Remote Sens. Spat. Inf. Sci. XL-7/W3, 557–562 (2015). These data are visible from an Indonesian government portal at <http://geoportal.menlhk.go.id/arcgis/rest/services/KLHK_EN>	These data include descriptions of described as: primary and secondary dryland forest, primary and secondary mangrove forest, primary and secondary swamp forest, plantation forest, bush/scrub, estate crop plantations, settlements areas, barren land, grass land, swamp, swamp shrub, dryland agriculture, scrub-mixed dryland farm, rice field, fish pond, post and harbour, transmigration areas, mining areas and open swamp. For the purposes of this study, settlement and mining areas were extracted specifically and separated for further spatial analyses. See also UA-C below.
M-A-B: Maus et al. (2020)	Polygon	Ca. 2019	Maus, V., Giljum, S., Luckeneder, S. (2020) A global-scale data set of mining areas. Scientific Data 7.<br>Maus, V., Giljum, S., Gutschlhofer, J., da Silva, D.M., Probst, M., Gass, S.L.B., Luckeneder, S., Lieber, M., McCallum, I., (2020) Global-scale mining polygons (Version 1). PANGAEA.	Polygons of coal mine areas ostensibly mapped with a 10km buffer. Total mine areas across the province are consequently ~8% greater than both M-A-A and M-A-C.
M-A-C: Sonter et al. (2020)	Polygon	Ca. 2015	Sonter, L.J., Simmonds, J.S., Watson, J.E., Jones, J.P., Kiesecker, J.M., Costa, H.M., Bennun, L., Edwards, S., Grantham, H.S., Griffiths, V.F. (2020) Local conditions and policy design determine whether ecological compensation can achieve No Net Loss goals. Nature Communications 11, 1-11.	Polygons of coal mine areas citing equal sources to M-A-A, although representing multiple time periods according to an author-derived land use model. Results as at 2015 were applied to the spatial overlays for analyses (3), (6), (8), (10) and (11).

S4.4 Human Settlements

Table S5: Summary of spatial data on villages as point locations (VP) and urban areas as polygons or rasters (UA)

ID	Data type	Date relevant	Source	Data Summary, Notes and Features.
**Village Points (VP)**				
V-P-A: OpenStreetMap	Point	2019-09	The files in this archive have been created from OpenStreetMap data and are licensed under the Open Database 1.0 License. This file contains OpenStreetMap data as of 2019-09-17T20:15:02Z. A documentation of the layers in this shape file is available at <http://download.geofabrik.de/osm-data-in-gis-formats-free.pdf>	Total N = 1144<br><br>City N = 3, hamlet N = 26, island N = 30, locality N = 1, region N = 1, town N = 8, village N = 1075<br><br>For purposes of analysis, we used only "village" data points as they constituted approx. 94% of total data points in number, and other categories appear not to reflect specific settlement areas. Population is listed for some data points, but not enough to warrant further analysis.
V-P-B: Indonesia Populated Places	Point	Downloaded 18/09/2019. From ArcGIS Online. Original date of data acquisition unknown.	Used as a layer for estimating village locations. The data description via the ArcGIS online portal cites <https://mapcruzin.com/free-indonesia-country-city-place-shapefiles.htm> as the original source.	Total N = 1043<br><br>City N = 4, hamlet N – 8, Island N = 30, region N = 7, state N = 1, suburb N = 1, town N = 4, village N – 983, "Yes" N = 5<br><br>For purposes of analysis, we used only "village" data points. These also constituted approx. 94% of total data points in number, and other categories appear not to reflect specific settlement areas. Population is listed only for some city data points.
V-P-C: Author sourced	Point	2020	Originally sourced via The Nature Conservancy Inodnesia in 2015, updated via visual inspection.	Total N = 816<br>All data points are villages, with each village (desa) individually named and independently verified.
**Village / Settlement Areas (VA)**				
VA-A: Global Urban Footprint	Raster	2013-14	Global Urban Footprint – accessed via <https://geoservice.dlr.de/web/maps/eoc:guf:3857>, August 2020. See:<br><br>Esch, T., Elsayed, S., Marconcini, M., Marmanis, D., Zeidler, J., Dech, S. (2014): Dimensioning the Degree of Urbanization – A Technical Framework for the Large-scale Characterization of Human Settlement Forms and Patterns based on Spatial Network Analysis. Submitted to Journal of Applied Geography.<br><br>Esch, T., Marconcini, M., Felbier, A., Roth, A., Heldens, W., Huber, M., Schwinger, M., Taubenböck, H., Müller, A., Dech, S. (2013): Urban Footprint Processor – Fully Automated Processing Chain Generating Settlement Masks from Global Data of the TanDEM-X Mission. IEEE Geoscience and Remote Sensing Letters, Vol. 10, No. 6, pp. 1617-1621. http://dx.doi.org/10.1109/LGRS.2013.2272953.<br><br>Esch, T., Taubenböck, H., Roth, A., Heldens, W., Felbier, A., Thiel, M., Schmidt, M., Müller, A., Dech, S. (2012): TanDEM-X Mission - New Perspectives for the Inventory and Monitoring of Global Settlement Patterns. Journal of Applied Remote Sensing, Vol. 6, No. 1, 061702 (October 04, 2012); 21 pp. http://dx.doi.org/10.1117/1.JRS.6.061702.<br><br>Esch, T., Schenk, A., Ullmann, T., Thiel, M., Roth, A. and S. Dech (2011): Characterization of Land Cover Types in TerraSAR-X Images by Combined Analysis of Speckle Statistics and Intensity Information. IEEE Transactions on Geoscience and Remote Sensing. Vol. 49, No.6, pp 1911 – 1925. http://dx.doi.org/10.1109/TGRS.2010.2091644.<br><br>Esch, T., Thiel, M., Schenk, A., Roth, A., Muller, A. and Dech, S. (2010): Delineation of Urban Footprints From TerraSAR-X Data by Analyzing Speckle Characteristics and Intensity Information. IEEE Transactions on Geoscience and Remote Sensing, Vol. 48, No. 2, pp. 905-916. http://dx.doi.org/10.1109/TGRS.2009.2037144<br>..	This dataset provides worldwide mapping of settlements with a high spatial resolution of 0.4 arcsec (~12 m). This dataset was cropped to the boundary of East Kalimantan. Further information available via <https://www.dlr.de/eoc/en/desktopdefault.aspx/tabid-9628/16557_read-40454/>
VA-B: GHS Built-up area<br><br>Temporal extent: 2016<br><br>20 m resolution.	Raster	2016	GHS Built-up area grid (GHS-BUILT), derived from Sentinel-1 (2016), R2018A [GHS_BUILT_S1NODSM_GLOBE_R2018A]. This product was distributed as part of the Community preRelease of the GHSL Data Package 2018 (GHS CR2018) (Florczyk et al. 2018);<br><br>Florczyk, A., Ehrlich, D., Corbane, C., Freire, S., Kemper, T., Melchiorri, M., Pesaresi, M., Politis, P., Schiavina, M., and Zanchetta, L. (2018a). Community pre-Release of GHS Data Package (GHS CR2018) in support to the GEO Human Planet Initiative. Publications Office of the European Union.	Florczyk, Aneta; Corbane, Christina; Schiavina, Marcello; Pesaresi, Martino; Maffenini, Luca; Melchiorri, Michele; Politis, Panagiotis; Sabo, Filip; Freire, Sergio; Ehrlich, Daniele; Kemper, Thomas; Tommasi, Pierpaolo; Airaghi, Donato; Zanchetta, Luigi (2019): GHS-UCDB R2019A - GHS Urban Centre Database 2015, multitemporal and multidimensional attributes. European Commission, Joint Research Centre (JRC) [Dataset]. Dataset describes the "human presence in the planet". Datasets indicate the presence of human populations and/or built-up infrastructure. PID: http://data.europa.eu/89h/53473144-b88c-44bc-b4a3-4583ed1f547e
UA-C: Indonesian Government Land Cover Data – Settlements	Vector	2013, 2014, 2015, 2017	Land cover and land use change datasets derived from the Ministry of Environment and Forestry (KLHK 2018), based on Landsat interpretation described in Wijaya, A. et al. (2015). Assessment of large-scale land cover change classifications and drivers of deforestation in Indonesia. ISPRS - Int. Arch. Photogramm. Remote Sens. Spat. Inf. Sci. XL-7/W3, 557–562 (2015). These data are visible from an Indonesian government portal at <http://geoportal.menlhk.go.id/arcgis/rest/services/KLHK_EN>	These data include descriptions of described as: primary and secondary dryland forest, primary and secondary mangrove forest, primary and secondary swamp forest, plantation forest, bush/scrub, estate crop plantations, settlements areas, barren land, grass land, swamp, swamp shrub, dryland agriculture, scrub-mixed dryland farm, rice field, fish pond, post and harbour, transmigration areas, mining areas and open swamp. For the purposes of this study, settlement and mining areas were extracted specifically and separated for further spatial analyses.
UA-D: Land cover spatial simulation modelling, East Kalimantan, 2015-2040	Raster	2015-2040	Sonter LJ, Simmonds JS, Watson JE, Jones JP, Kiesecker JM, Costa HM, et al. Local conditions and policy design determine whether ecological compensation can achieve No Net Loss goals. Nature Communications 2020; 11: 1-11.	Land cover raster dataset based on UA-C, but further assessed and modelled to consider potential changes to 2040.

S5. Additional figures, tables, and detailed results

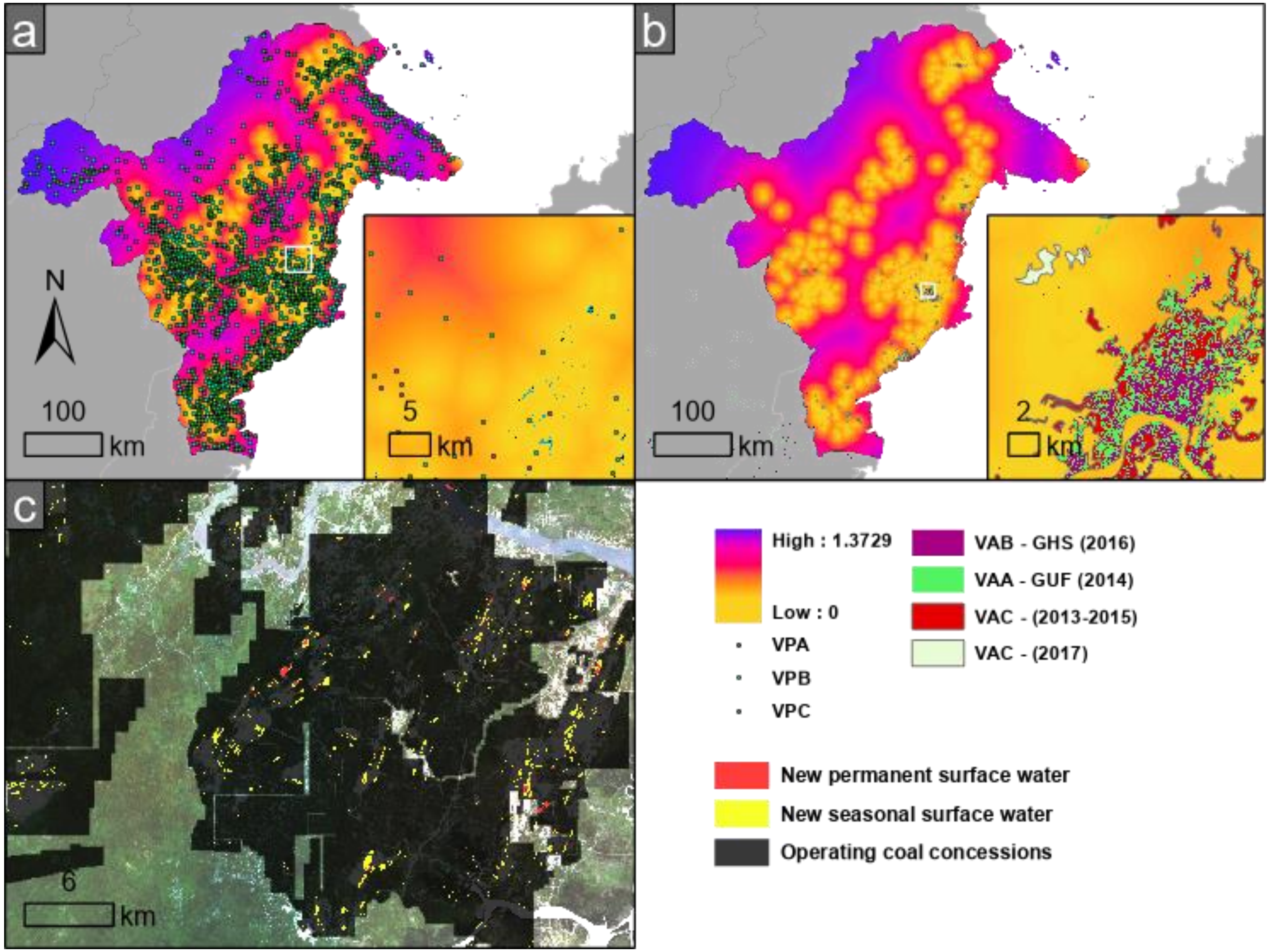

Fig S7: (a) Village point locations relative to proximity to coal pit water bodies; (b) Human settlement (raster or polygon) locations and spread relative to proximity to coal pit water bodies; (c) Coal pit water bodies emerged in operating coal concessions (south Samarinda area). Euclidean distance raster has units of decimal degrees.

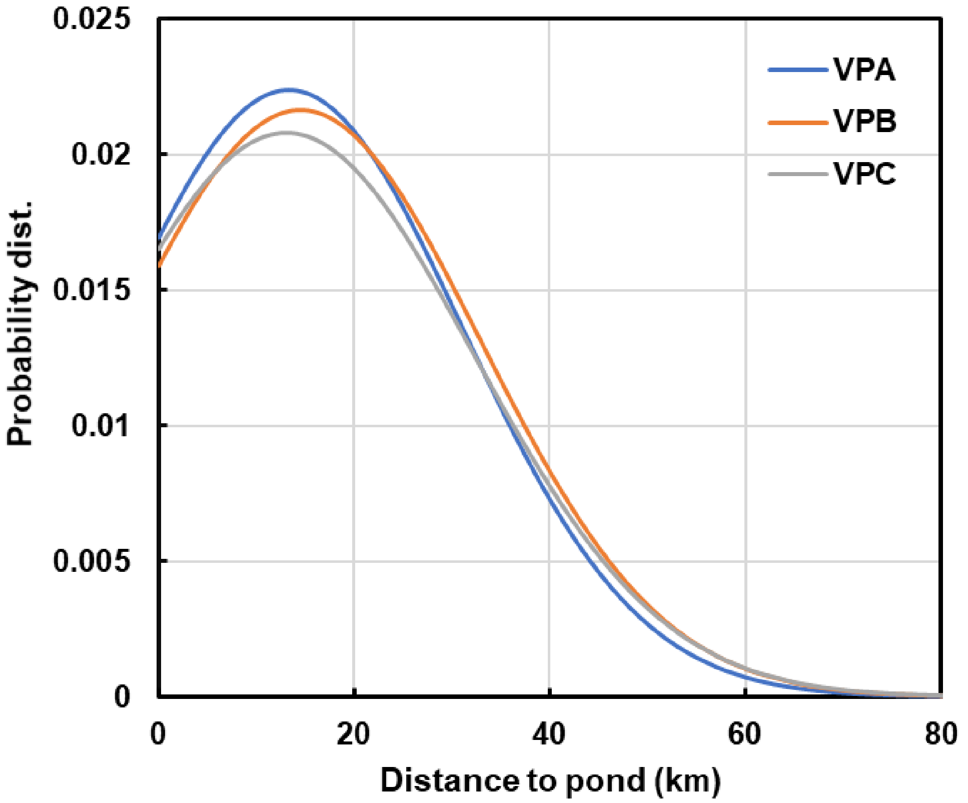

Figure S8: Overlay (1) - Village point locations with respect to their distance to pit ponds in operating coal concessions. While each dataset contains different numbers and locations of village centres, they collectively produce large agreement on the co-location of villages and coal water ponds. VPA/VPB/VPC refer to village point datasets per Table S5.

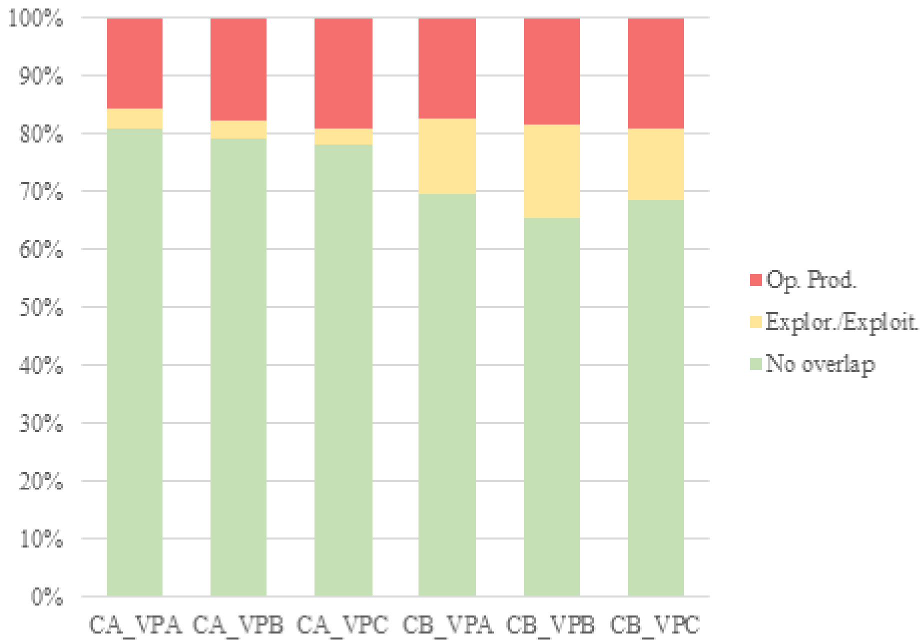

Figure S9: Overlay (2) – Village / settlement point data in relation to coal concessions, disaggregated by the operating status of the concessions. VPA/VPB/VPC refer to village point datasets per Table S5. CA/CB refer to concession datasets per Table S3.

Table S5: Overlay (4) – Village / Settlement polygon data in relation to their proximity to water bodies. Used Zonal Statistics in ArcGIS Pro to identify the overlap between a Euclidean distance raster of distances to coal ponds. For each settlement area polygon, the Min, Max, Range, Mean and Standard deviation of distance values are summarised (noting that a polygon can show multiple distances to a pond area depending on the edges measured).

Overlay	Min (km)	Max (km)	Min – Avg (km)	Max – Avg (km)	Range -Avg (km)	Mean – Avg (km)	St. Dev. -Avg (km)
WB v VAA (Global Urban Footprint) N = 11,366 polygons.	0	128.79	7.15	7.25	0.01	7.201	0.03
WB v VAB (Global Human Settlements) N = 25594 polygons.	0	104.97	5.70	5.73	0.02	5.72	0.01
WB v VAC (Global Urban Footprint) N = 643 polygons.	0	70.64	8.95	9.90	0.95	9.42	0.24

Table S6: Overlay (5) – Intersections between urban / settlement area polygons and mine permit areas (VA v C). N represents the number of polygons intersecting with each permit or non-permit data category. Overall average settlement area overlapping coal permits = 14.2%, σ = 3.6%.

**Global Human Settlement v JATAM Concessions (VAAvCA)**	Area (ha)	N =
Total Settlement Area Outside Concessions	26093.77	12661
Area inside concessions: PKP2B - Operating	2349.37	2378
Area inside concessions: PKP2B - Exploration	202.08	202
Area inside concessions: IUP - Operating	2179.43	2549
Area inside concessions: IUP - Exploration	252.66	211
% Total settlement area overlapping coal	**16%**	

**Global Human Settlement v The Nature Conservancy Concessions (VAAvCB)**	Area (ha)	N =
Total Settlement Area Outside Concessions	24897.29	12045
Area inside concessions: Operating	4734.04	5945
Area inside concessions: Exploration	1032.75	1573
Area inside concessions: Exploitation	0.09	1
Area inside concessions: Backup	8.45	5
Area inside concessions: Under investigation	-	-
% Total settlement area overlapping coal	**19%**	

**Global Human Settlement v EDSM Concessions (VAAvCC)**	Area (ha)	N =
Total Settlement Area Outside Concessions	26571.59	13080
Area inside concessions: PKP2B - Operating	2392.32	2372
Area inside concessions: PKP2B - Exploration	0.02	2
Area inside concessions: IUP - Operating	2112.19	2526
Area inside concessions: IUP - Exploration	-	-
% Total settlement area overlapping coal	**14%**	

Table S6: Overlay (5) (cont.)

**Global Urban Footprint v JATAM Concessions (VABvCA)**	Area (ha)	N =
Total Settlement Area Outside Concessions	7542.53	24411
Area inside concessions: PKP2B - Operating	497.36	2926
Area inside concessions: PKP2B - Exploration	25.37	176
Area inside concessions: IUP - Operating	224.15	1754
Area inside concessions: IUP - Exploration	78.69	270
% Total settlement area overlapping coal	**10%**	

**Global Urban Footprint v The Nature Conservancy Concessions (VABvCB)**	Area (ha)	N =
Total Settlement Area Outside Concessions	7378.96	23770
Area inside concessions: Operating	791.14	5878
Area inside concessions: Exploration	198.58	1108
Area inside concessions: Exploitation	0.04	2
Area inside concessions: Backup	0.35	5
Area inside concessions: Under investigation	0.12	2
% Total settlement area overlapping coal	**12%**	

**Global Urban Footprint v EDSM Concessions (VABvCC)**	Area (ha)	N =
Total Settlement Area Outside Concessions	7662.92	24922
Area inside concessions: PKP2B - Operating	499.85	2900
Area inside concessions: PKP2B - Exploration	-	-
Area inside concessions: IUP - Operating	206.42	1697
Area inside concessions: IUP - Exploration	-	-
% Total settlement area overlapping coal	**8%**	

Table S6: Overlay (5) (cont.)

**Indonesia Government Land Cover v JATAM Concessions (VACvCA)**	Area (ha)	N =
Total Settlement Area Outside Concessions	50049.37	531
Area inside concessions: PKP2B - Operating	5313.83	103
Area inside concessions: PKP2B - Exploration	156.93	25
Area inside concessions: IUP - Operating	2926.82	134
Area inside concessions: IUP - Exploration	483.85	4
% Total settlement area overlapping coal	**15%**	

**Indonesia Government Land Cover v The Nature Conservancy Concessions (VACvCB)**	Area (ha)	N =
Total Settlement Area Outside Concessions	47844.43	837
Area inside concessions: Operating	9138.35	1293
Area inside concessions: Exploration	1947.99	145
Area inside concessions: Exploitation	-	-
Area inside concessions: Backup	-	-
Area inside concessions: Under investigation	-	-
% Total settlement area overlapping coal	**19%**	

**Indonesia Government Land Cover v EDSM Concessions (VACvCC)**	Area (ha)	N =
Total Settlement Area Outside Concessions	50541.43	542
Area inside concessions: PKP2B - Operating	5329.39	120
Area inside concessions: PKP2B - Exploration	-	-
Area inside concessions: IUP - Operating	3059.98	138
Area inside concessions: IUP - Exploration	-	-
% Total settlement area overlapping coal	**14%**	

Table S7: Overlay (11) – Intersections between mine areas and coal concession (permit) areas (MA v C). Values below represent the count and area of polygons situated within and outside different permit categories. Average area outside of permits = 17920 ha (σ = 2753 ha), with an average count of 231 violation instances (σ = 44). Note that concession types (left column) are described further in Table S1. MAA = Indonesia Gov't Land Cover Data (2013-2017), MAB = Mine Area Polygons from Maus et al. (2020), MAC = Mine areas per Sonter et al. (2020). CA = JATAM Concession data, CB = The Nature Conservancy Concession data, CC = EDSM Concession data.

**MAAvCA**				
	Operating Production	Exploration	Exploitation	Outside
IUP (Count)	386	5		
IUP (Total ha)	35643.67	24.70		
PKP2B (CCoW) (Count)	215			
PKP2B (CCoW) (Total ha)	78917.67			
KK (Count)			3	
KK (Total ha)			737.16	
Outside (Count)				259
Outside (Total ha)				15617.08
TOTAL Count	601	5	3	259
TOTAL ha	114561.34	24.70	737.16	15617.08
		% of Mine areas outside permit areas:		**11.9%**

**MABvCA**			
	Operating Production	Exploration	Outside
IUP (Count)	422	4	
IUP (Total ha)	38344.38	306.55	
PKP2B (CCoW) (Count)	248	1	
PKP2B (CCoW) (Total ha)	85508.93	0.55	
Outside (Count)			233
Outside (Total ha)			16148.37
TOTAL Count	670	5	233
TOTAL ha	123853.32	307.10	16148.37
	% of Mine areas outside permit areas:		**11.5%**

MACvCA				
	Operating Production	Exploration	Exploitation	Outside
IUP (Count)	394	4		
IUP (Total ha)	31706.82	21.16		
PKP2B (CCoW) (Count)	279	1		
PKP2B (CCoW) (Total ha)	69949.05	0.22		
KK (Count)			5	
KK (Total ha)			734.43	
Outside (Count)				257
Outside (Total ha)				14704.04
TOTAL Count	673	5	5	257
TOTAL ha	101655.8649	21.38	734.43	14704.04
	% of Mine areas outside permit areas:			**12.6%**

MAAvCB					
	Operating Production	Exploration	Exploitation	No status	Outside
Count	1260	76	8	418	190
Total ha	115936.98	1348.91	265.96	7064.04	6009.85
		% of Mine areas outside permit areas:			**4.6%**

MABvCB						
	Operating Production	Exploration	Exploitation	No status	Other non-cnc concession (Not explicitly marked as Batubara)	Outside
Count	1305	74	1	5	425	162
Total ha	124856.14	1326.11	194.34	231.42	8619.27	5452.19
			% of mine areas outside permit areas:			**3.9%**

MACvCB						
	Operating Production	Exploration	Exploitation	No status	Other non-cnc concession (Not explicitly marked as Batubara)	Outside
Count	1347	75	1	7	410	175
Total ha	103517.60	1482.32	11.90	312.46	6485.55	4999.58
			% of mine areas outside permit areas:			**4.3%**

MAAvCC		
	Operating Production	Outside
IUP (Count)	378	
IUP (Total ha)	33767.15	
PKP2B (CCoW) (Count)	196	
PKP2B (CCoW) (Total ha)	76483.76	
Outside (Count)		277
Outside (Total ha)		21058.09
TOTAL Count	574	277
TOTAL ha	110250.91	21058.09
	% of Mine areas outside permit areas:	**16.0%**

MABvCC		
	Operating Production	Outside
IUP (Count)	425	
IUP (Total ha)	37483.42	
PKP2B (CCoW) (Count)	246	
PKP2B (CCoW) (Total ha)	83589.49	
Outside (Count)		247
Outside (Total ha)		20562.63
TOTAL Count	671	247
TOTAL ha	121072.92	20562.63
% of Mine areas outside permit areas:		**14.5%**

**MACvCC**		
	Operating Production	Outside
IUP (Count)	388	
IUP (Total ha)	30130.56	
PKP2B (CCoW) (Count)	262	
PKP2B (CCoW) (Total ha)	67556.02	
Outside (Count)		277
Outside (Total ha)		19431.75
TOTAL Count	650	277
TOTAL ha	97686.58	19431.75
	% of Mine areas outside permit areas:	**16.6%**

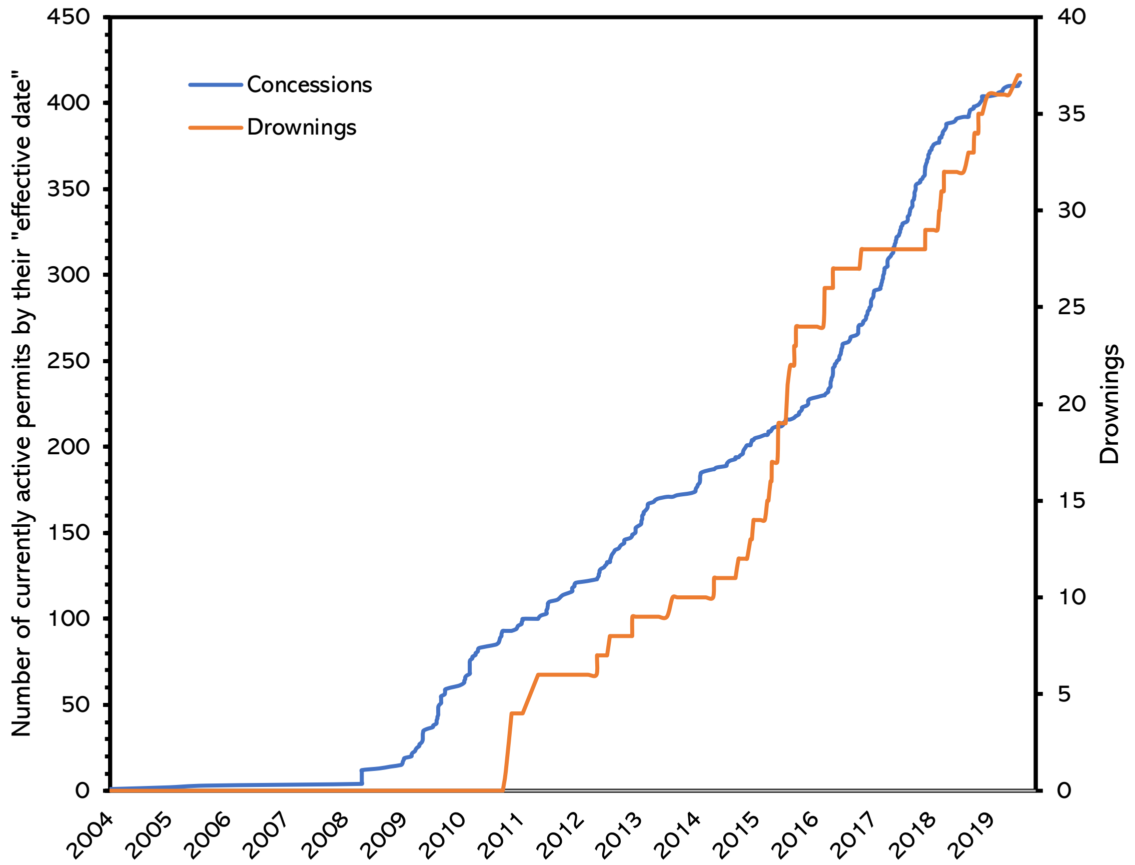

Figure S10: Increase in the number of concessions (established by the effective date listed in the C-C Concession dataset) versus the increase in drownings over the same time period 2008-2019. Establishes a temporal link between the presence of coal concession areas and drownings in East Kalimantan, supplementing the spatial link otherwise shown in this study.

429 **Table S8: Compiled records of victims of non-remediated mine pits in East Kalimantan. Names have been removed here for sensitivity, but are publicly reported in the sources identified.**

Number (Age of victim, if known)	Company	Location	Date of death	Lat	Long	Location Accuracy[1]	Notes
1. (10)	PT Hymco Coal	Sungai Kerbau Kecamatan Sambutan, Samarinda	12/07/2011	- 0.51737	117.1712	H	Location obtained from: Kholis, N.; Laila, S. N.; Aswidah, R.; Haryono, O.; Surya, D.; Cahyani; Suntoro, A.; Arimurti, A. N.; Johansyah, M.; Agustiorini, S.; Sari, M.; Hariyadi, D.; Jari, T.; Saini, A. THE VIOLATION OF BASIC RIGHTS IN THE CASE OF FORMER MINING PIT IN EAST KALIMANTAN; National Commision on Human Rights, (Komisi Nasional Hak Asisi Manusia): 2016.
2. (13)	PT Hymco Coal	Sungai Kerbau Kecamatan Sambutan, Samarinda	12/07/2011	- 0.51737	117.1712	H	Location obtained from: Kholis, N.; Laila, S. N.; Aswidah, R.; Haryono, O.; Surya, D.; Cahyani; Suntoro, A.; Arimurti, A. N.; Johansyah, M.; Agustiorini, S.; Sari, M.; Hariyadi, D.; Jari, T.; Saini, A. THE VIOLATION OF BASIC RIGHTS IN THE CASE OF FORMER MINING PIT IN EAST KALIMANTAN; National Commision on Human Rights, (Komisi Nasional Hak Asisi Manusia): 2016.
3.	PT Hymco Coal	Sungai Kerbau Kecamatan Sambutan, Samarinda	12/07/2011	- 0.51737	117.1712	H	Location obtained from: Kholis, N.; Laila, S. N.; Aswidah, R.; Haryono, O.; Surya, D.; Cahyani; Suntoro, A.; Arimurti, A. N.; Johansyah, M.; Agustiorini, S.; Sari, M.; Hariyadi, D.; Jari, T.; Saini, A. THE VIOLATION OF BASIC RIGHTS IN THE CASE OF FORMER MINING PIT IN EAST KALIMANTAN; National Commision on Human Rights, (Komisi Nasional Hak Asisi Manusia): 2016.
4. (6)	PT Panca Prima Mining	Sambutan Idaman Permai, Pelita 2, Samarinda	24/12/2011	- 0.50748	117.2198	L	Searching for this location gives the location of a group of housing adjacent to some open pit areas, but the location itself seems to be the name of the housing, rather than a description of the village/suburb. The location selected represents the largest pit adjacent to these buildings.
5. (6)	PT Panca Prima Mining/PD. PAU	Sambutan Idaman Permai, Pelita 2, Samarinda	24/12/2011	- 0.50748	117.2198	L	Searching for this location gives the location of a group of housing adjacent to some open pit areas, but the location itself seems to be the name of the housing, rather than a description of the village/suburb. The location selected represents the largest pit adjacent to these buildings.
6.	PT Kitadin	Tenggarong Seberang, Kukar	2011	- 0.32486	117.0976	M	Location of mine determined from Government concession data. Pit area to the north-west of the specified point is used.
7. (14)	PT Kitadin	Desa Bangun Rejo Kecamatan Tenggarong Seberang, Kukar	26/01/2016	- 0.35882	117.1227	M	Correct concession identified. Selected a central pit.
8. (11)	PT Insani Bara Perkasa	Blok B RT 20, Simpang Pasir, Palaran, Samarinda	24/12/2012	- 0.5592	117.1507	H	Location obtained from: Kholis, N.; Laila, S. N.; Aswidah, R.; Haryono, O.; Surya, D.; Cahyani; Suntoro, A.; Arimurti, A. N.; Johansyah, M.; Agustiorini, S.; Sari, M.; Hariyadi, D.; Jari, T.; Saini, A. THE VIOLATION OF BASIC RIGHTS IN THE CASE OF FORMER MINING PIT IN EAST KALIMANTAN; National Commision on Human Rights, (Komisi Nasional Hak Asisi Manusia): 2016.
9. (8)	Tak teridentifikasi	Sambutan, Pelita 4, Handil Kopi, Blok L No 4, Samarinda	14/03/2013	- 0.50893	117.1876	L	Possible small-scale, illegal mining. Location approximated to waste rock adajcent to the reported streets.
10. (11)	PT Muliana Jaya	Kelurahan Jawa RT. 4 Kecamatan Sangasanga, Kukar	08/2013	- 0.6638	117.2344	M	Central pit within the correct concession area selected. Precise pit unknown.
11 (10)	PT Energi Cahaya Industritama	Kelurahan Rawa Makmur, Kecamatan Palaran, Samarinda	8/04/2014	- 0.58451	117.1799	H	Location obtained from: Kholis, N.; Laila, S. N.; Aswidah, R.; Haryono, O.; Surya, D.; Cahyani; Suntoro, A.; Arimurti, A. N.; Johansyah, M.; Agustiorini, S.; Sari, M.; Hariyadi, D.; Jari, T.; Saini, A. THE VIOLATION OF BASIC RIGHTS IN THE CASE OF FORMER MINING PIT IN EAST KALIMANTAN; National Commision on Human Rights, (Komisi Nasional Hak Asisi Manusia): 2016.
12. (10)	PT Graha Benua Etam	Sempaja, Samarinda	22/12/2014	- 0.41896	117.1814	H	Location obtained from: Kholis, N.; Laila, S. N.; Aswidah, R.; Haryono, O.; Surya, D.; Cahyani; Suntoro, A.; Arimurti, A. N.; Johansyah, M.; Agustiorini, S.; Sari, M.; Hariyadi, D.; Jari, T.; Saini, A. THE VIOLATION OF BASIC RIGHTS IN THE CASE OF FORMER MINING PIT IN EAST KALIMANTAN; National Commision on Human Rights, (Komisi Nasional Hak Asisi Manusia): 2016.
13. (13)	PT Cahaya Energi Mandiri	Kelurahan Sambutan, Samarinda	23/05/2015	- 0.49165	117.2209	H	Location obtained from: Kholis, N.; Laila, S. N.; Aswidah, R.; Haryono, O.; Surya, D.; Cahyani; Suntoro, A.; Arimurti, A. N.; Johansyah, M.; Agustiorini, S.; Sari, M.; Hariyadi, D.; Jari, T.; Saini, A. THE VIOLATION OF BASIC RIGHTS IN THE CASE OF FORMER MINING PIT IN EAST KALIMANTAN; National Commision on Human Rights, (Komisi Nasional Hak Asisi Manusia): 2016.
14. (14)	PT Bara Sigi Mining (BSM)	Desa Sebulu Modern, RT 14, (jalan poros Tenggarong-Sebulu), Kecamatan Sebulu, Kukar	5/08/2015	- 0.30862	116.9814	M	Drowned in a former mining pit owned by PT Cakra at Sebulu Modern Village, District Sebulu, Regency of Kutai Kartanegara. Location is to the nearest pit in this concession.
15. (11)	PT Lana Harita Indonesia	Sungai Siring, Samarinda	24/08/2015	- 0.40548	117.2467	H	Location obtained from: Kholis, N.; Laila, S. N.; Aswidah, R.; Haryono, O.; Surya, D.; Cahyani; Suntoro, A.; Arimurti, A. N.; Johansyah, M.; Agustiorini, S.; Sari, M.; Hariyadi, D.; Jari, T.; Saini, A. THE VIOLATION OF BASIC RIGHTS IN THE CASE OF FORMER MINING PIT IN EAST KALIMANTAN; National Commision on Human Rights, (Komisi Nasional Hak Asisi Manusia): 2016.
16. (12)	PT Transisi Energi Satunama	Lok Bahu, Samarinda	18/11/2015	- 0.4982	117.0751	H	Location obtained from: Kholis, N.; Laila, S. N.; Aswidah, R.; Haryono, O.; Surya, D.; Cahyani; Suntoro, A.; Arimurti, A. N.; Johansyah, M.; Agustiorini, S.; Sari, M.; Hariyadi, D.; Jari, T.; Saini, A. THE VIOLATION OF BASIC RIGHTS IN THE CASE OF FORMER MINING PIT IN EAST KALIMANTAN; National Commision on Human Rights, (Komisi Nasional Hak Asisi Manusia): 2016.
17. (16)	CV Atap Tri Utama	Bantuas, Palaran Samarinda	8/12/2015	- 0.55246	117.2054	H	Location obtained from: Kholis, N.; Laila, S. N.; Aswidah, R.; Haryono, O.; Surya, D.; Cahyani; Suntoro, A.; Arimurti, A. N.; Johansyah, M.; Agustiorini, S.; Sari, M.; Hariyadi, D.; Jari, T.; Saini, A. THE VIOLATION OF BASIC RIGHTS IN THE CASE OF FORMER MINING PIT IN EAST KALIMANTAN; National Commision on Human Rights, (Komisi Nasional Hak Asisi Manusia): 2016.
18. (15)	PT Multi Harapan Utama (MHU)	RT 3, Kelurahan Loa Ipuh Darat, Kecamatan Tenggarong, Kukar	16/12/2015	- 0.46133	116.8534	M	Nearest pit to the village marker selected. Within PT Multi Harapan Utama (MHU) concession
19. (9)	KSU Wijaya Kusuma	Desa Sumber sari Kecamatan Sebulu, Kukar	30/12/2016	- 0.18777	117.0053	H	Location obtained from: Kholis, N.; Laila, S. N.; Aswidah, R.; Haryono, O.; Surya, D.; Cahyani; Suntoro, A.; Arimurti, A. N.; Johansyah, M.; Agustiorini, S.; Sari, M.; Hariyadi, D.; Jari, T.; Saini, A. THE VIOLATION OF BASIC RIGHTS IN THE CASE OF FORMER MINING PIT IN EAST KALIMANTAN; National Commision on Human Rights, (Komisi Nasional Hak Asisi Manusia): 2016.
20. (20)	PT Bumi Energi Kaltim	Buluminung, Penajam, Penajam Paser utara	12/02/2016	- 1.24866	116.6493	H	Location obtained from: Kholis, N.; Laila, S. N.; Aswidah, R.; Haryono, O.; Surya, D.; Cahyani; Suntoro, A.; Arimurti, A. N.; Johansyah, M.; Agustiorini, S.; Sari, M.; Hariyadi, D.; Jari, T.; Saini, A. THE VIOLATION OF BASIC RIGHTS IN THE CASE OF FORMER MINING PIT IN EAST KALIMANTAN; National Commision on Human Rights, (Komisi Nasional Hak Asisi Manusia): 2016.
21. (15)	PT Bukit Baiduri Energi	Desa Bukit Raya RT. 19 Kecamatan Tenggarong Seberang, Kukar	23/03/2016	- 0.42209	117.0721	H	Location obtained from: Kholis, N.; Laila, S. N.; Aswidah, R.; Haryono, O.; Surya, D.; Cahyani; Suntoro, A.; Arimurti, A. N.; Johansyah, M.; Agustiorini, S.; Sari, M.; Hariyadi, D.; Jari, T.; Saini, A. THE VIOLATION OF BASIC RIGHTS IN THE CASE OF FORMER MINING PIT IN EAST KALIMANTAN; National Commision on Human Rights, (Komisi Nasional Hak Asisi Manusia): 2016.
22. (15)	PT Bukit Baiduri Energi	Desa Bukit Raya RT. 19 Kecamatan Tenggarong Seberang, Kukar	23/03/2016	- 0.42209	117.0721	H	Location obtained from: Kholis, N.; Laila, S. N.; Aswidah, R.; Haryono, O.; Surya, D.; Cahyani; Suntoro, A.; Arimurti, A. N.; Johansyah, M.; Agustiorini, S.; Sari, M.; Hariyadi, D.; Jari, T.; Saini, A. THE VIOLATION OF BASIC RIGHTS IN THE CASE OF FORMER MINING PIT IN EAST KALIMANTAN; National Commision on Human Rights, (Komisi Nasional Hak Asisi Manusia): 2016.
23.	PT Insani Bara Perkasa	Palaran, Samarinda	9/04/2016	- 0.58418	117.1406	M	Location determined to be within the Palaran portion of the PT Insani Baraperkasa concession. Note: Was exposed to burning coal. Died 28 days later after 6 operations.
24. (17)	PT Insani Bara Perkasa	KM. 9, RT 18, Desa Purwajaya, Kecamatan Loa Janan, Kukar	15/05/2016	- 0.65737	117.0941	M	Cross–referenced the correct concession with the reported village. Selected the largest pit area within the areas that crosses both.
25. (22)	CV Panca Bara Sejahtera	Samarinda Ulu, Samarinda	3/05/2016	- 0.44325	117.1407	H	Location obtained from: Kholis, N.; Laila, S. N.; Aswidah, R.; Haryono, O.; Surya, D.; Cahyani; Suntoro, A.; Arimurti, A. N.; Johansyah, M.; Agustiorini, S.; Sari, M.; Hariyadi, D.; Jari, T.; Saini, A. THE VIOLATION OF BASIC RIGHTS IN THE CASE OF FORMER MINING PIT IN EAST KALIMANTAN; National Commision on Human Rights, (Komisi Nasional Hak Asisi Manusia): 2016.
26. (15)	PT Energy Cahaya Industritama	Bukuan, Palaran, Samarinda	8/11/2016	- 0.59607	117.2141	M	Largest pit in the PT Energi Cahaya concession selected.
27. (15)	PT Energy Cahaya Industritama	Bukuan, Palaran, Samarinda	8/11/2016	- 0.59607	117.2141	M	Largest pit in the PT Energi Cahaya concession selected.
28. (18)	PT Gunung Bayan Pratama Coal	Pit 7D5, Desa Belusuh, Kecamatan Siluq Ngurai, Kutai Barat	25/06/2017	- 0.55866	115.9468	M	Although the pit number is specified, detailed mine layout information could not be obtained to identify this. The largest pit of the concession was used.
29. (25)	CV Prima Coal Mining	Lubang CV.Prima Coal Mining, Samarinda	27/07/2018	-	-	N/A	Location could not be determined, as the authors could not identify a CV Prima Coal concession within the Samarinda region when cross-checking against other concession data.

30. (16)	PT Trias Patriot Sejahtera	Desa Rapak Lambur (Mangkurawang), Tenggarong, Kukar	21/10/2018	-0.33055	116.9669	M	Location of mine determined from Government concession data.
31. (13)	PT Bukit Baiduri Energi	Desa Bukit Raya, Kecamatan Tenggarong Seberang, Kukar	4/11/2018	-0.41828	117.08	M	Location determined to be a pit within the area overlapping the reported village (Bukit Raya) and the reported concession (owned by PT Bukit Baiduri Energi)
32. (10)	Illegal Mining	Rapak Dalam, Samarinda Seberang, Samarinda	20/11/2018	-0.51682	117.1385	L	Illegal mining operation. Selected the only water body visible within the village boundary that is not a part of any legal concession.
33. (14)	PT Mandala Usaha Tambang Utama	Desa Bunga Jadi, Kecamatan Muara Kaman, Kutai Kartanegara	21/04/2019	-0.17584	116.8913	M	Selected the only pit clearly visible within the reported village border, and cross-checked against point location data from government concession sources.
34.	PT Insani Bara Perkasa	Jalan Kebon Agung, Rt.12 Kelurahan Simpang Pasir, Kecmatan Palaran, Samarinda	29/05/2019	-0.56604	117.1362	M	Selected a pit adjacent to the street of the reported incident, within the company's concession boundary.
35. (10)	PT Insani Bara Perkasa	Kelurahan Bukit Pinang Gg.Saka RT.16 No.100, Kecamatan Samarinda Ulu, Samarinda	22/06/2019	-0.45236	117.0974	M	Nearest road and concession known. Pit nearest to the road within this concession selected.
36. (25)	PT Singlurus Pratama	Desa Beringin Agung, Kecamatan Samboja, Kabupaten Kutai Kartenegara	22/08/2019	-0.951287	117.0837	H	Exact location reported in: <https://www.mongabay.co.id/2019/09/02/korban-jiwa-di-lubang-tambang-masalah-besar-ibu-kota-baru-indonesia/>, accessed 2/06/2020
37. (21)	PT Cahaya Energi Mandiri (CEM)	Jalan Kalan Luas, Lubuk Sawah, Samarinda , East Kalimantan	23/02/2020	-0.48234	117.2208	M	Selected pit adjacent to Jalan Kalan Luas within the PT Cahaya Energi Mandiri concession boundary.
38. (14)	PT Sarana Daya Hutama (SDH)	Krayan Makmur village, Paser district, East Kalimantan	6/09/2020	-1.63784	116.2397	H	Exact location reported in: <https://news.mongabay.com/2020/09/indonesia-mine-pits-paser-east-kalimantan-accident-death-sarana-daya-hutama/>
39.	PT Sarana Daya Hutama (SDH)	Krayan Makmur village, Paser district, East Kalimantan	7/09/2020	-1.63784	116.2397	H	Exact location reported in: <https://news.mongabay.com/2020/09/indonesia-mine-pits-paser-east-kalimantan-accident-death-sarana-daya-hutama/>

[1]High location accuracy (H) means a precise location, to the nearest pit, has been determined from news articles and/or published reports. Medium (M) means the correct concession is identified, with a central or major pit within this concession selected. Usually this involves cross-referencing the reported village boundary with the boundary of the concession, and/or identifying pits nearest to the reported road on which the incident occurred. Low (L) means that authors could not identify the correct concession area, or that the incident took place outside of a legal concession (possibly due to the incident occurring in an informal mining area), in which case a pit was selected nearest to the reported address, within the reported village boundary.

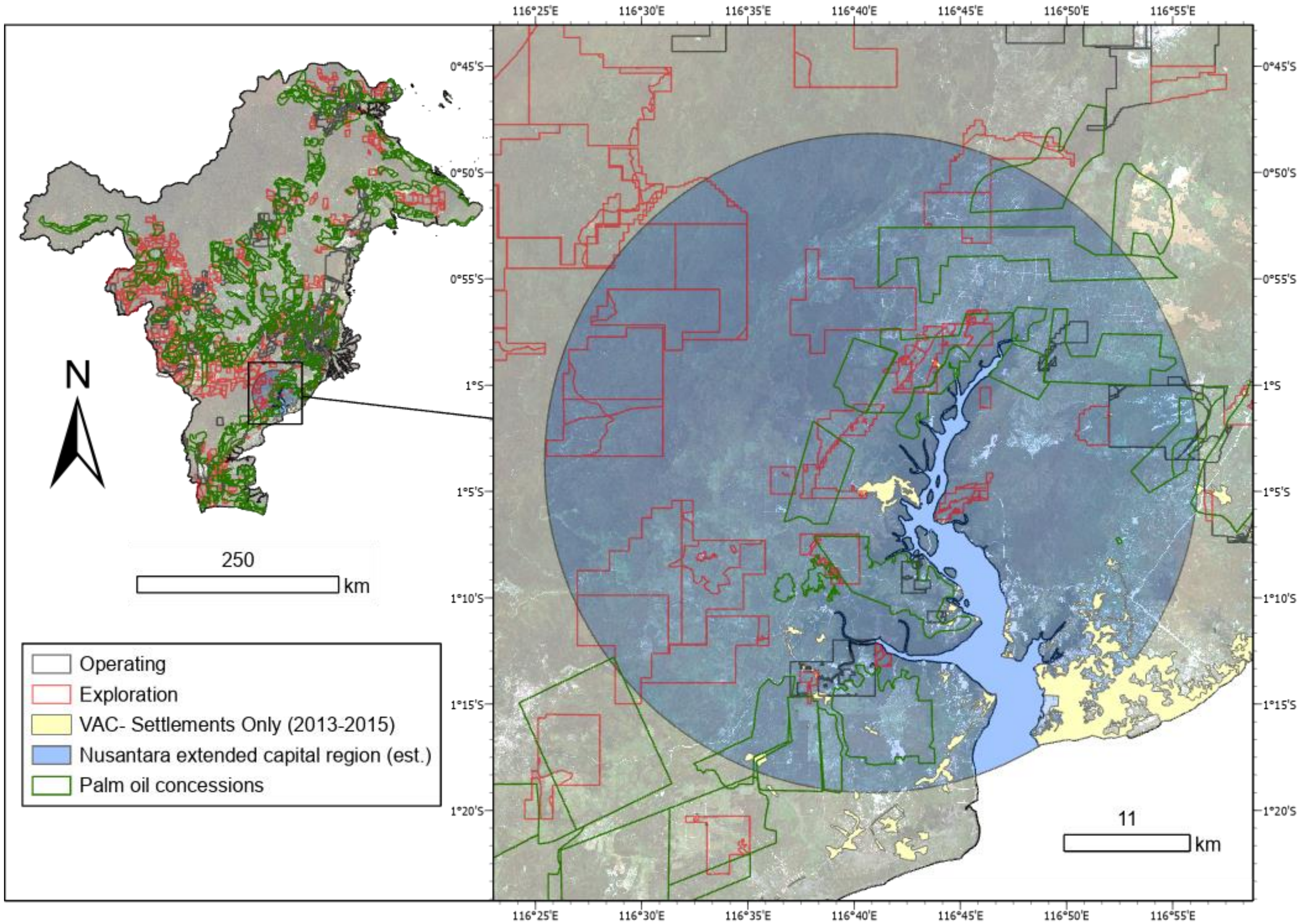

Figure S11: Overlap of the extended Nusantara capital region against coal exploration areas, and existing palm oil concessions. Extended capital region based on an approximation of the area and centre location as reported in the Draft Laws Regarding the National Capital (Naskah Akademik Rancangan Undang-Undang Tentang Ibu Kota Negara) – see <https://bappeda.kaltimprov.go.id/storage/data-centers/September2021/zNPFAwFfhrKe6NOUadXI.pdf>. Actual future administrative boundaries are unclear, however this overlay indicates considerable overlap with areas under consideration for coal and oil palm activities. It remains to be seen to what extent the new city will permit such activities to coexist to same extent as has been shown in East Kalimantan's current capital Samarinda.

Supplementary References

Atteridge, A., Aung, M.T., Nugroho, A., 2018. Contemporary coal dynamics in Indonesia. Stockholm Environment Institute.

Butt, S., Lindsey, T., 2018. Indonesian law. Oxford University Press, Oxford.

Daulay, B., 1994. Tertiary coal belt in easter Kalimantan, Indonesia: the influence of coal quality on coal utilisation. University of Wollongong, Wollongong, Australia.

Devi, B., 2013. Mining and Development in Indonesia: An Overview of the Regulatory Framework and Policies. Sustainable Minerals Institute, The University of Queensland, Centre for Social Responsibility in Mining.

Dudley, B. 2019. BP Statistical Review of World Energy. *BP Statistical Review.* 68th ed. London, United Kingdom.

Erb, M., Mucek, A.E., Robinson, K., 2021. Exploring a social geology approach in eastern Indonesia: What are mining territories? Extr. Ind. Soc. 8, 89–103. https://doi.org/10.1016/j.exis.2020.09.005

Esch, T., Marconcini, M., Marmanis, D., Zeidler, J., Elsayed, S., Metz, A., Müller, A., Dech, S., 2014. Dimensioning urbanization–An advanced procedure for characterizing human settlement properties and patterns using spatial network analysis. Applied Geography 55, 212-228.

Florczyk, A.C., Christina; Schiavina, Marcello; Pesaresi, Martino; Maffenini, Luca; Melchiorri, Michele; Politis, Panagiotis; Sabo, Filip; Freire, Sergio; Ehrlich, Daniele; Kemper, Thomas; Tommasi, Pierpaolo; Airaghi, Donato; Zanchetta, Luigi 2019. GHS-UCDB R2019A - GHS Urban Centre Database 2015, multitemporal and multidimensional attributes, in: European Commission, J.R.C.J. (Ed.).

Fox, J., Adhuri, D., Resosudarmo, I.A.P., 2005. Unfinished edifice or Pandora’s box? Decentralisation and resource management in Indonesia, in: Resosudarmo, B.P. (Ed.), The Politics and Economics of Indonesia’s Natural Resources. Institute of Southeast Asian Studies, Singapore.

Friederich, M.C., van Leeuwen, T., 2017. A review of the history of coal exploration, discovery and production in Indonesia: The interplay of legal framework, coal geology and exploration strategy. Int. J. Coal Geol. 178, 56–73. https://doi.org/10.1016/j.coal.2017.04.007

Funfgeld, A., 2018. Just energy? Structures of energy (in)justice and the Indonesian coal sector, in: Jafry, T., Mikulewicz, M., Helwig, K. (Eds.), Routledge Handbook of Climate Justice. Routledge.

IEA, 2020. Coal 2020: Analysis and forecast to 2025. International Energy Agency, Paris.

Kiswanto, Tsuyuki, S., Mardiany, Sumaryono, 2018. Completing yearly land cover maps for accurately describing annual changes of tropical landscapes. Global Ecology and Conservation 13, e00384.

Maus, V., Giljum, S., Gutschlhofer, J., da Silva, D.M., Probst, M., Gass, S.L.B., Luckeneder, S., Lieber, M., McCallum, I., 2020a. Global-scale mining polygons (Version 1). PANGAEA.

Maus, V., Giljum, S., Luckeneder, S., 2020b. A global-scale data set of mining areas. Scientific Data 7.

Mietzner, M., 2007. Party Financing in Post-Soeharto Indonesia: Between State Subsidies and Political Corruption. Contemp. Southeast Asia 29, 238–263.

Nugroho, H., 2019. Indonesia's Energy Development: Evaluation of the 2015-2019 Medium Term Development Plan and Outlook for that of 2020-2024. J. Perenc. Pembang. Indones. J. Dev. Plan. 3, 266–272. https://doi.org/10.36574/jpp.v3i3.87

OCallaghan, T., 2010. Patience is a virtue: Problems of regulatory governance in the Indonesian mining sector. Resour. Policy 35, 218–225. https://doi.org/10.1016/j.resourpol.2010.05.001

OECD, 2019. OECD Green Growth Policy Review of Indonesia 2019, OECD Environmental Performance Reviews. OECD. https://doi.org/10.1787/1eee39bc-en

Pekel, J.-F., Cottam, A., Gorelick, N., Belward, A.S., 2016. High-resolution mapping of global surface water and its long-term changes. Nature 540, 418-422.

Phan, T.N., Kuch, V., Lehnert, L.W., 2020. Land Cover Classification using Google Earth Engine and Random Forest Classifier—The Role of Image Composition. Remote Sensing 12, 2411.

PwC Indonesia, 2019. Mining in Indonesia: Investment and Taxation Guide. PricewaterhouseCoopers.

Robinson, K., 2016. Mining, land and community rights in Indonesia, in: McCarthy, J.F., Robinson, K. (Eds.), Land and Development in Indonesia: Searching for the People's Sovereignty. ISEAS-Yusof Ishak Institute.

Sonter, L.J., Simmonds, J.S., Watson, J.E., Jones, J.P., Kiesecker, J.M., Costa, H.M., Bennun, L., Edwards, S., Grantham, H.S., Griffiths, V.F., 2020. Local conditions and policy design determine whether ecological compensation can achieve No Net Loss goals. Nature Communications 11, 1-11.

Toumbourou, T., Muhdar, M., Werner, T., Bebbington, A., 2020. Political ecologies of the post-mining landscape: Activism, resistance, and legal struggles over Kalimantan's coal mines. Energy Res. Soc. Sci. 65, 101476. https://doi.org/10.1016/j.erss.2020.101476

Velentina, R.A., 2020. Legal certainty for foreign investors in coal mining in Indonesia. J. Huk. Pembang. 49, 923. https://doi.org/10.21143/jhp.vol49.no4.2349

Wijaya, A., Budiharto, R. S., Tosiani, A., Murdiyaso, D. & Verchot, L. V. 2015. Assessment of large scale land cover change classifications and drivers of deforestation in Indonesia. *The International Archives of Photogrammetry, Remote Sensing and Spatial Information Sciences*, 40**,** 557.
